# MtSNPscore: a combined evidence approach for assessing cumulative impact of mitochondrial variations in disease

**DOI:** 10.1186/1471-2105-10-S8-S7

**Published:** 2009-08-27

**Authors:** Anshu Bhardwaj, Mitali Mukerji, Shipra Sharma, Jinny Paul, Chaitanya S Gokhale, Achal K Srivastava, Shrish Tiwari

**Affiliations:** 1 Genomics and Molecular Medicine, Institute of Genomics and Integrative Biology, CSIR, Mall Road, Delhi 110007, India; 2 Max Planck Institute for Evolutionary Biology, August-Thienemann-Str. 2, 24306 Plön, Germany; 3 Neuroscience Centre, All India Institute of Medical Sciences, New Delhi 110029, India; 4 Bioinformatics Division, Centre for Cellular and Molecular Biology, CSIR, Uppal Road, Hyderabad 500007, India

## Abstract

**Background:**

Human mitochondrial DNA (mtDNA) variations have been implicated in a broad spectrum of diseases. With over 3000 mtDNA variations reported across databases, establishing pathogenicity of variations in mtDNA is a major challenge. We have designed and developed a comprehensive weighted scoring system (MtSNPscore) for identification of mtDNA variations that can impact pathogenicity and would likely be associated with disease. The criteria for pathogenicity include information available in the literature, predictions made by various *in silico *tools and frequency of variation in normal and patient datasets. The scoring scheme also assigns scores to patients and normal individuals to estimate the cumulative impact of variations. The method has been implemented in an automated pipeline and has been tested on Indian ataxia dataset (92 individuals), sequenced in this study, and other publicly available mtSNP dataset comprising of 576 mitochondrial genomes of Japanese individuals from six different groups, namely, patients with Parkinson's disease, patients with Alzheimer's disease, young obese males, young non-obese males, and type-2 diabetes patients with or without severe vascular involvement. MtSNPscore, for analysis can extract information from variation data or from mitochondrial DNA sequences. It has a web-interface  that provides flexibility to update/modify the parameters for estimating pathogenicity.

**Results:**

Analysis of ataxia and mtSNP data suggests that rare variants comprise the largest part of disease associated variations. MtSNPscore predicted possible role of eight and 79 novel variations in ataxia and mtSNP datasets, respectively, in disease etiology. Analysis of cumulative scores of patient and normal data resulted in Matthews Correlation Coefficient (MCC) of ~0.5 and accuracy of ~0.7 suggesting that the method may also predict involvement of mtDNA variation in diseases.

**Conclusion:**

We have developed a novel and comprehensive method for evaluation of mitochondrial variation and their involvement in disease. Our method has the most comprehensive set of parameters to assess mtDNA variations and overcomes the undesired bias generated as a result of better-studied diseases and genes. These variations can be prioritized for functional assays to confirm their pathogenic status.

## Background

Mitochondria are the primary energy-generating organelles in eukaryotes possessing the oxidative phosphorylation system (OXPHOS) comprised of five protein complexes. While the majority of the protein subunits of these complexes are nuclear encoded a set of 13 protein subunits as well as 2 rRNAs and 22 tRNAs are encoded in human mitochondrial DNA (mtDNA) [1]. These form the essential structural and functional components of complexes I, III, and IV of the electron transport chain and of complex V (ATP synthase). Besides, mitochondria are also involved in other processes like intracellular signalling, apoptosis and intermediary metabolism [2].

Mitochondrial dysfunction leading to disease phenotypes with diverse and over-lapping symptoms as well as multi-organ involvement is being increasingly reported. These result from mutations in mtDNA or nuclear genes and in a majority of cases typically have cardiac and neurological manifestations [3-5]. Heritability of mitochondrial diseases is highly variable – ranging from maternal, to Mendelian to a combination of the two [2]. The presence of both heteroplasmic and homoplasmic mtDNA along with extensive basal polymorphisms of the mitochondrial genome (over 3000 variations are reported across databases like OMIM [6], MitoMap [7] and mtDB [8]) further complicate the genetic analysis of mtDNA diseases.

Establishing pathogenicity of a sequence change in mtDNA or identifying causal/functional polymorphisms from this huge diversity remains a major challenge despite many attempts in this direction [9]. For instance, the canonical criteria for pathogenic mtDNA point mutations proposed by DiMauro and Schon [10] is limited by its dependence on the presence of heteroplasmy, a feature which is not universal for pathogenicity [9]. Efforts toward determining pathogenicity for the tRNA [11] and Complex I genes [9], using various criteria are still insufficient for classification of a large proportion of reported mutations or to predict their impact on phenotype and fitness [10].

There is growing body of evidence that mitochondrial dysfunction plays a crucial role in the pathogenesis of or influences the risk of diseases, such as Alzheimer's, Parkinson's, cardiovascular disease including cardiomyopathy, etc [3,4,12]. But the exact role and involvement of mtDNA mutations in causing these diseases is unclear and debatable [13,14]. This prompted us to develop a comprehensive method for assessing pathogenic impact of mtDNA variations. This novel method, MtSNPscore, identifies and scores disease associated mtDNA variations by filtering out polymorphic sites and sites with no reported or predicted functional role. It also provides a cumulative score for the entire mitochondrial genome in the patient and normal individual. Thus it allows prioritization of variations which could significantly impact function as well as predicts through cumulative analysis whether mitochondrial associated pathogenesis could be implicated in a diseased individual.

This method has been tested on variations in 92 sporadic ataxia patients whose mtDNA was sequenced in this study. Ataxia is a central nervous system (CNS) manifestation etiologically characterized by heterogeneous symptoms e.g. myoclonus or action tremor, sensory loss, pyramidal signs, etc. This disorder is generally associated with varying length of repeat expansion in the nuclear genome. However, in most populations nearly 50% of the cases of ataxia are sporadic and are not associated with repeat expansion. In one of the autosomal recessive ataxias, Friedreich's ataxia, involvement of the frataxin gene encoding a mitochondrial protein has been demonstrated. This suggests that mitochondria may have an important role in the etiology of ataxia [15]. These sporadic cases provide an ideal test set for exploring the role of mitochondrial variation in ataxia. Involvement of trinucleotide repeat expansion in all the known ataxia genes was excluded in the studied individuals.

We also analyzed mtSNP data comprising of 576 mitochondrial genomes of Japanese individuals from six different groups, namely, patients with Parkinson's disease, Alzheimer's disease, young obese males, young non-obese (thin) males, and type-2 diabetes patients with or without severe vascular involvement and 96 Japanese centenarians as controls [16].

## Results and discussion

### Identification and integration of pathogenicity parameters for scoring mtDNA variations

**Figure 1:**
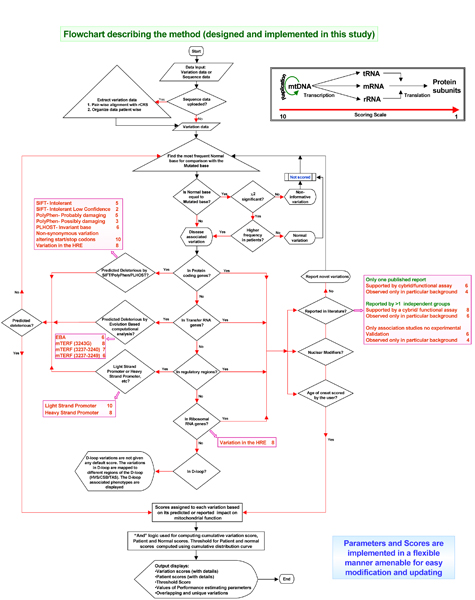
**Flowchart describing the method (designed and implemented in this study) – displays summary of Weighted Scores (WS)**. The flowchart depicts that input can either be variation or sequence data. In case sequence data is provided, it is converted to variation data. Although the variation data is generated by comparison with rCRS, the most common normal base is used to compare the frequency of the variation in patients and normal individuals. The variation is scanned for reported or predicted functional impact only if its frequency is higher in patients or the variation is absent in the normal individuals. Each variation is tested for its reported or predicted functional impact based on its genomic context (in protein coding genes/tRNA/rRNA, etc). A Weighted Score is assigned to each variation based on the information obtained from literature and the *in silico *prediction tools (scores shown in pink boxes). As seen in the inset, the scoring scheme is conceptually driven following the Central Dogma and the scores range from 1 to 10, where a more deleterious variation gets a higher score. The parameters and scores are implemented in a flexible manner to ensure their easy modification and updation with the advent of new information.

The MtSNPscore pipeline [Figure 1] starts with extraction of single base changes from mitochondrial DNA sequences. We have used the Revised Cambridge Reference Sequence (rCRS- GenBank: AC_000021, gi: 115315570) as the reference sequence for this purpose. A variation is scored for pathogenicity only if it is unique to patients or its frequency is significantly higher in patients compared to normal individuals. Each variation is assigned a Weighted Score (WS) based on its predicted/reported functional impact. The scoring scheme is conceptually based on the central dogma. Since mitochondrial depletion has been reported in many diseases, the factors that regulate mtDNA replication are thus given the highest scores. Further, the absence of protein subunits has also been implicated in various mitochondrial diseases and hence transcription initiation and regulatory sites are also given high scores. Changes at conserved sites in the members of the translation machinery (tRNAs and rRNAs) are expected to be deleterious and are scored depending on their conservation and implication in diseases based on literature survey and through computational predictions. Missense mutations are also analyzed for their effect on protein structure/function using various prediction tools (details in Materials and methods).

### Validation of *in silico *prediction tools incorporated in MtSNPscore

Four *in silico *prediction methods have been incorporated in our scoring scheme to assess variations for their functional significance and possible role in disease etiology. The efficacy of these methods was tested using a dataset of "reported pathogenic mutations" from OMIM, MitoMap and PubMed. Mutations which fulfilled at least one of the following criteria were included in the analysis-

(a) a reported biochemical effect (for example, lowered activities of the complexes)

(b) is supported by functional studies in cell lines (trans-mitochondrial cybrids)

(c) have been independently reported in multiple studies

As a first step, we selected mutations from literature. 121 of them which satisfied the above-mentioned criteria were integrated in the pipeline [Additional file 1]. Of these, 52 mutations were in protein-coding genes, 66 in tRNA and three in rRNA genes [Additional file 2].

Mutations in the protein-coding genes were used for testing the predictive power of SIFT [17], PolyPhen [18], PHD-SNP [19] and PLHOST [20]). Excluding mutations leading to six stop codons and three INDELs the remaining 43 mutations were tested by the four methods.

All the mutations were predicted to be deleterious/intolerant/disease-associated by at least one of the prediction methods, 30 by two, 16 by three and two mutations by all the four methods [Additional file 3 and Additional file 4]. Pathogenic mutations in the tRNA genes analyzed using the compensatory co-evolution method [21] predicted 54 out of the 66 reported tRNA gene mutations to be deleterious. Of the remaining 12 mutations in tRNA genes, majority were in the loop region and were not predicted to be deleterious by this method [Additional file 3]. Taking the above observations into consideration, we integrated all of these tools into our pipeline to comprehensively assess the functional impact of novel mutations.

### Frequency difference is not an effective parameter to associate variations in mitochondria to diseases or phenotypes

**Figure 2:**
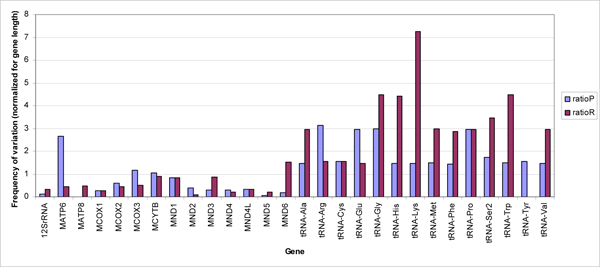
**Normalized distribution of prioritized variation across mtDNA**. The graph shows a gene-wise comparison of reported (ratioR) and high scoring (ratioP) variations normalized for gene length. It can be seen that there is a bias towards tRNA genes which are also reported hot spots in many mitochondrial diseases.

Amongst all the variations, those which had relatively higher weighted score and hence predicted to be potentially deleterious were designated as prioritized variation in ataxia and mtSNP. We observed these variations in 28 of the 37 mitochondrial genes in both the data sets [Figure 2]. A majority of these were in tRNA genes which are also reported hot spots in many mitochondrial diseases.

**Figure 3:**
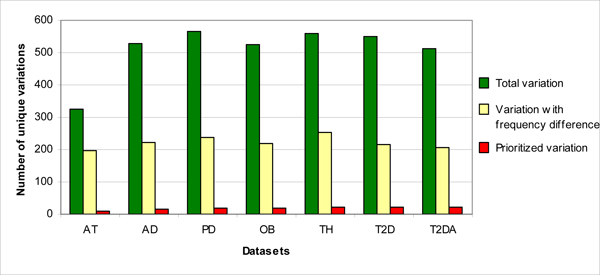
**Summary of prioritized variation compared to total variation**. Frequency difference does not seem to be a sole determinant for disease association. It can be seen that almost 50% (Yellow) of the total variation (Green) in all the datasets differ in frequency between normal individuals and patients. On the contrary, inclusion of other information prioritized only 3–4% (Red) of the total variation.

**Figure 4:**
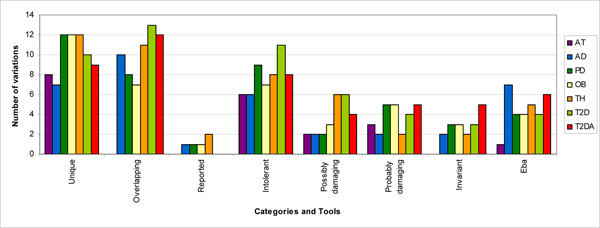
**Summary of results – showing properties of prioritized variations in Ataxia and mtSNP groups**. The summary of the output generated by our method for the ataxia patients and mtSNP diseases. As is clear from the graph, some variations are prioritized in more than one dataset but some are unique to the phenotypes, represented as 'overlapping' and 'unique', respectively. Thus, these unique variations are still more potent targets for designing functional assays. All variations prioritized in Ataxia are unique. Further, of the total 92 prioritized variations only 5 have been reported. (Intolerant – Amino acids changes predicted deleterious by SIFT; Possibly/Probably damaging – Amino acids changes predicted deleterious by PolyPhen; Invariant – Present at invariant sites as predicted by PLHOST; Eba – tRNA variations Predicted deleterious by compensatory co-evolution method; AT – Ataxia; AD – Alzheimer's Disease; PD – Parkinson's Disease; T2DA – Type2 Diabetes With Angiopathy; T2D – Type2 Diabetes; OB – Obese; TH – Thin).

Comparison of all observed variations in normal and patient datasets show that almost half of these variations occur with high frequency in patients [Figure 3], whereas a much smaller fraction is predicted to be potentially deleterious. Thus, frequency difference does not seem to be a sole determinant for disease association. A large number of variations when analysed in the pipeline seem to be inconsequential as determined by the weighted scores. We discuss these observations in seven datasets – Ataxia and six mtSNP phenotypes [Figure 4].

### Ataxia data analysis

We compared entire mitochondrial genome sequences from 92 ataxia patients against a control dataset comprised of 92 sequences from Indian population retrieved from mtDB [8]. Variations were observed at 324 positions across the mitochondrial genome in these ataxia patients. Some of the variations were common and present in equal frequencies in normal individuals and patients and some (197) were observed at a higher frequency in ataxia cases. Even though more that 50% of the variations were observed at high frequency in the patients, only eight variations gave a WS greater than the threshold value set at 3 [The threshold for WS has been detailed in the methods section]. Amongst these seven (1 – MT-ND3; 1 – MT-ND5; 2 – MT-COX1; 1 – MT-COX3; 2 – MT-ATP6) map to the protein-coding genes and one to the MT-TR gene [Additional file 5]. The T10191C mutation in MT-ND3 is reported to be associated with Complex I deficiency [22,23]. This non-synonymous mutation changes a hydrophilic serine to hydrophobic proline and it is possible that defects in protein-folding may lead to decreased enzymatic activity. In ataxia, we observed T10191G (the mutant base is G and not C, as reported), which changes serine to alanine, another hydrophobic residue. This variation is absent from the Indian control dataset and is also predicted to be 'Intolerant' by SIFT. This combined information lends credence to the proposed deleterious effect of this mutation. Thus, from an initial set of 324 variations we prioritized 8 potentially disease-associated candidates. However, functional assays are required to confirm their pathogenic status.

### mtSNP data analysis

To assess the involvement of mitochondrial mutations in other diseases, we analyzed with our method mtSNP [16] data which consists of entire mitochondrial genome sequences of seven groups from Japanese diseased and healthy population. The Japanese Centenarians were used as control data for the analysis. Our method predicted 3–4% of the total variations (WS >= 3) as disease associated for each mtSNP group. In Alzheimer's disease (AD), from a total of 529 variations, 17 high-scoring variations were obtained. Similarly, for Parkinson's disease (PD) – 20/567, Type 2 diabetes with angiopathy (T2DA) – 21/513, Type 2 diabetes without angiopathy (T2D) – 23/550, obese phenotype (OB) – 19/525 and thin phenotype (TH) – 23/558 were assigned high WSs [Additional file 5; Figure 3; Figure 4]. These high scoring variants are probably disease-associated. Out of the total of 84 putatively pathogenic variants, five have been reported to be disease associated in various studies.

We also observed a few overlapping high scoring variations across mtSNP datasets not observed in the centenarians. This suggests the systemic involvement of these mitochondrial variations in diseases or phenotypes [Figure 4]. For instance, 10 out of 17 high scoring variations in Alzheimer's dataset are also scored high in other mtSNP phenotypes indicating a modifier role for these variations. The seven variations not observed in other sets may be unique to Alzheimer's and can be tested for validation in different AD cohorts.

Overall statistics of the mtSNP data are similar to the trends obtained from ataxia subset. From 1275 non-redundant sites which vary in the mitochondrial genome across these datasets our method prioritized 84. Only five of these 84 have been reported to be disease associated in various studies. Thus the remaining 79 high scoring variants distributed across protein-coding (some of which are highly conserved amino acids in mammals), tRNA, 12SrRNA genes and the D-loop can be potential candidates associated with mtSNP phenotypes. Our method is not biased towards prioritizing known mtDNA variations and thus demonstrates that integration of available information with predictions would be helpful in identifying candidates for disease association studies and to some extent overcomes the ascertainment bias resulting from better-studied genes and diseases.

### Cumulative scoring from entire mtDNA hints at mitochondrial involvement in disease

MtSNPscore also assigns cumulative scores to patients and normal individuals. This allows assessment of the overall impact of variations. In the case of the Ataxia patients, the score ranges from 1 to 41.2 and from a total of 92 patients, 32 scored more than the threshold score. On the other hand, none of the 92 normal individuals had a score higher than the threshold score. This resulted in an MCC value of 0.46 and accuracy of 0.67. Similar values were computed for the mtSNP datasets where the MCC values range from 0.5 to 0.55 and accuracy from 0.7 to 0.73 [Additional file 6]. This suggests that the approach is useful in differentiating normal from patients and is particularly significant in case of mitochondrial diseases where it is difficult to assess the disease-causing role of mitochondrial variations in the presence of enormous amount of background variations.

### Limitations and future strategies

Our method is an effort to provide a conceptual framework for prioritization of variations in mtDNA to assess their functional involvement in disease phenotypes. We have tested our hypothesis on a limited dataset restricted to a few studies. Mitochondrial variations can be tissue specific or its function could be a consequence of mutations in nuclear DNA, or variability in environment factors. Also, there are various studies wherein mitochondrial variations associated to disease phenotypes are observed only in muscle tissue but not in blood [24]. Therefore any inferences on study of mitochondrial variations from peripheral blood have to be made with caution.

Since the field of mitochondrial genomics has gained considerable importance leading to a surge in sequencing data [25,26] our method is designed in a flexible manner to incorporate any novel changes in the analysis pipeline. We expect that the performance of this method will improve in future updates with the advent of additional literature and a widespread production of standardized genome-wide association data. As a prospect, this can also be enhanced as a systems approach wherein detrimental defects in any one of the genes encoding members of a correlated set of reactions i.e., reactions that always or often function together in metabolic network, can result in similar phenotypic consequences. This approach will further help in understanding the genotype-phenotype relationships and the potential identification of therapeutic targets and strategies for disease treatment [27].

Nuclear modifiers are also known to play an important role in mitochondrial diseases and hence are also accounted for in the scoring scheme. For example, a mutation in the MT-TE (T14709C) is reported to alter an evolutionarily conserved nucleotide in its anti-codon loop (reference in Additional file 1) and is expected to be deleterious. In this context, another study [28] suggests the involvement of nuclear factors in the expression of the phenotype. This indicates that nuclear background is significant while assessing the effect of certain mitochondrial mutations. However, this feature is presently not scored in MtSNPscore. Also, in the present study D-loop mutations were not scored in the datasets and the pipeline only maps and reports the D-loop mutations. Given the enormous heterogeneity in frequency of different variants in this region, it is difficult to assess these variations for disease association. This issue cannot be resolved by comparing allele frequency differences among cases and controls but requires understanding of the world phylogeny of human mtDNA [29]. However, MtSNPscore web server may be easily customized to score D-loop mutations for further analysis.

Our scoring scheme priorities only mtDNA mutations, however, it is important to note that there are 170 nuclear-encoded OXPHOS subunits and an estimated 1,500 mitochondrial proteins that are as yet untested. A systematic evaluation of mtDNA and nucleus-encoded mitochondrial genes and an investigation of epistatic interactions between the two genomes will reveal the extent to which mitochondrial defects play a causal role in human disease.

## Conclusion

We have developed a novel and comprehensive method to prioritize mtDNA variations for disease association. It is evident from the analysis of sporadic ataxia patients and mtSNP datasets, rare variants comprise a bulk of the high-scoring mutations. Our results indicate that common variants in mitochondrial genome are not disease associated as also corroborated by earlier reports [14,30]. Therefore, whole mitochondrial genome sequencing is imperative to evaluate its involvement in a particular disease or phenotype instead of studying the effect of individual variations. Overall, MtSNPscore even in this limited dataset has predicted 87 novel candidates. We believe that this approach can be implemented as a regular pre-screening method in high throughput entire mitochondrial genome sequencing studies to prioritize mtDNA mutations for disease association and to evaluate mitochondrial involvement in disease as well as in reducing subsequent genotyping cost and effort. These can be followed up through functional assays to confirm their pathogenic status.

## Materials and methods

### Datasets for analysis

The study was carried out on complete mitochondrial genome sequences of 92 probands diagnosed for sporadic ataxia and 92 (Indian) control data from mtDB [8]. Most of the patients were from Northern India and clinically diagnosed at the Neuroscience Department of All India Institute of Medical Sciences (AIIMS). Patients were clinically tested for neurological symptoms in legs, arms, tremor, etc. There was a wide heterogeneity in the age of onset ranging from 1 year to 74 years. The involvement of trinucleotide repeats expansion in all the known ataxia genes was excluded in all these samples [Additional file 7].

### Extraction of variation data from mtSNP database (patients)

#### Generation of variation data from sequences and *vice versa*

Variation information was extracted from mtSNP database  that provides the entire mitochondrial genome sequences of Japanese individuals belonging to six different groups, with 96 individuals in each group, namely, patients with Parkinson's disease, patients with Alzheimer's disease, young obese males, young non-obese males, and type-2 diabetes patients with or without severe vascular involvement. These sequences were aligned to the rCRS genes to generate variation data. A PERL module has been developed for alignment and precise mapping of variations, taking care of insertion/deletions in addition to substitutions. These variations were organized individual-wise. Often instead of complete sequences only variations are reported for the complete mitochondrial genome of an individual. In such cases to obtain the complete mitochondrial genome of the patient the variations are mapped onto the reference sequence. Another module has been developed to stitch the variation data on the background of the reference mitochondrial DNA to generate patient's mitochondrial genome. This module adjusts for INDELs (insertion-deletions) and accordingly generates the entire mitochondrial genome for the patient dataset. In human mtDNA there are seven instances of overlapping bases among genes [Additional file 8]. As can be seen that the maximum overlap is between MT-ATP6 and MT-ATP8 of 45 bases, followed by 6 base overlap between MT-ND4 and MT-ND4L, 3 base overlap between MT-TI and MT-TQ and four single base overlaps between MT-ND1/MT-TI, MT-TC/MT-TY, MT-CO1/MT-TS and MT-ATP6/MT-CO3. It is obvious that these variations when reported as per the rCRS will map to two different genes and hence care is taken to map and predict the impact of variations in such overlapping regions.

### Selecting control dataset

As mentioned earlier, for our ataxia patients, the control dataset (Indian) was taken from mtDB. Of the 672 Japanese sequences in mtDB, 96 sequences belonging to Japanese Centenarians were used as control dataset for comparison against the mtSNP datasets. This was done primarily because of absence of valid control groups in the mtDB database, as the remaining 576 Japanese sequences in mtDB are sequences of these mtSNP datasets. Also, Centenarians might have accumulated maximum possible mtDNA mutations with age but were still healthy. However, it is important to mention that the role of mitochondrial mutations in mammalian aging remains speculative, and it is still an area of intense debate [31]. For this study, Centenarians worked as a good control set, given that their age, phenotype and variations are known, as compared to selecting a random normal set where these parameters may not be available.

### Selection of predictive parameters and development of scoring scheme

#### Scoring reported mutations

Weighted Scores are assigned to each reported mutation as mentioned above [scores presented in Additional file 1, column 5; Figure 1 contains a summary of the Weighted Scores]. In case there is only one published report, the mutation is scored only when a cybrid (fused rho zero cells with donor cells from patients harbouring mtDNA mutations) or a functional assay supports the findings. The default score assigned for this category is 6. In case the mutation has been reported by more than one independent group and is supported by a cybrid or functional assay, it is assigned a score of 8. On the contrary, if multiple association studies have reported the mutation but no other experimental validation is available, then a score of 6 is assigned. However, if the mutant phenotype is observed only in a particular background (nuclear or mitochondrial), then 2 is subtracted from the scores [Figure 1].

#### Variation in regulatory sites

##### (i) Promoters

Mammalian mitochondrial genome contains 2 promoters: LSP (Light Strand Promoter) & HSP (Heavy Strand Promoter), which produce near-genomic length transcripts. After transcription RNA processing releases individual mRNAs, tRNAs and rRNAs [32]. Transcription from LSP is necessary not only for gene expression but also for production of RNA primers required for initiation of mtDNA replication. Mitochondrial polymerase recognizes promoter elements in a sequence-specific manner [33]. Mutations may change this interaction and affect the mtDNA transcription rate. Hence mutations in LSP and HSP are given default high scores of 10 and 8, respectively (score for LSP is higher because it plays dual role – in mtDNA replication and transcription).

##### (ii) Hormone Response Elements (HRE)

There are four HREs in the mitochondrial genome present in MT-RNR1, MT-RNR2 and MT-TL1, MT-ND1 and MT-DLOOP. A study suggests that steroid and thyroid hormone effects on the mitochondrial genes are direct, concomitant with the effects on nuclear genes and involving similar molecular mechanisms as those mediating steroid-thyroid hormone actions on nuclear gene transcription [34]. The variations in HREs may affect transcription, therefore these changes are given a high score of 8.

#### Variation in tRNA genes

Variations of the 22 mitochondrial tRNAs are of particular interest because these tRNAs span only 10% of the human mitochondrial genome yet they harbor more than half of all known mitochondrial pathogenic mutations. It has been reported that mitochondrial tRNA stem sites harboring pathogenic mutations have a tendency to co-evolve with their complementary stem sites and this can be used to distinguish pathogenic mutations from polymorphisms. On the basis of these criteria of conservation and compensatory co-evolution, a mutation can be classified as either benign or deleterious [21]. This method follows evolution based computational analysis of the differences between pathogenic and non-pathogenic substitutions. It has a high rate of accuracy for distinguishing benign variants from severely and slightly pathogenic ones. This method has been applied to all possible mutations of the 22 mitochondrial tRNAs. The mutations disrupting Watson-Crick (WC) pairing in stems were predicted to have the highest probability of being deleterious, whereas mutations in stems that to do not disrupt WC pairs have the lowest. Mutations that are not located in stems have an intermediate probability of being deleterious, most likely due to the inclusion of the highly conserved anticodon loop. Lethal mutations were also classified as deleterious by this approach. Our method integrates the results of this published analysis to score tRNA variations and assigns deleterious changes a score of 6.

Transcription termination of the human mitochondrial genome requires specific binding to termination factor, mTERF. mTERF binding site coincides with tRNA-Leu (UUR) [3237–3249]. Mutations that map within these 13 bases are given a score of 6. Further, a conserved fragment within this region (TGGC 3237–3240) is given a score of 7. Furthermore, it has been shown that at 3243 position, "G" is not tolerated, therefore this mutation is given a high score of 8. Thus, scoring for mTERF is categorized in three sections based on varying degrees of conservation within this fragment [35]. A recent report implicates mTERF in mtDNA replication as well [36], further underlining the importance of variations in this region.

#### Variation in rRNA genes

For ribosomal RNA, reported mutations and HRE sites are screened and scored if present in the patient variation data.

#### Variation in protein coding genes

Changes in protein coding genes can be synonymous or non-synonymous. Codon assignments for mtDNA are different from the universal genetic code and thus the alternate codon table is utilized for reporting codon changes [37]. For both non-synonymous and synonymous changes, scores are assigned based on their association with mitochondrial dysfunction as reported in literature. In addition, for non-synonymous changes, predictions from PolyPhen (Polymorphism Phenotyping: gives predictions on the basis of structure and function using physical and comparative parameters), SIFT (Sorting Intolerant from Tolerant: gives prediction based on amino acid conservation) and PLHOST (Peptide library based homology search tool) have been used. Predictions of PolyPhen and SIFT were obtained for the entire set of mitochondrial proteins by replacing the wild-type amino acid at all positions with every other amino acid. PolyPhen classifies a mutation as probably damaging, possibly damaging, benign or unknown. If a mutation is predicted to be probably damaging it is given a score of 5 and if possibly damaging it is given a score of 3. Similarly, SIFT classifies a mutation as tolerant or intolerant. A score of 5 is assigned to intolerant mutations with high confidence and 2 to intolerant mutations with low confidence. Further, mutations in protein motifs that are conserved across species are expected to be deleterious. Such invariant peptides have been predicted using PLHOST. Mutations mapping to invariant peptides are given a score of 6. Lastly, modulus of the difference in the hydrophobicity values of the normal and mutated amino acids (obtained from Wimley & White [38]) is added to the score. Non-synonymous mutations altering the initiation and stop codons are given a high score of 10.

A threshold score is set to differentiate between possible benign and candidate pathogenic variations. In MtSNPscore, the threshold weighted score is 3 as this is the lowest score assigned to any parameter indicating the pathogenicity of the variation.

### Segregating variations based on their frequency difference

Variation frequency in patients is compared against the control data by applying the Chi-square test. Although variation data is generated by comparison with the rCRS, for finding frequency difference, the most common base in the normal individuals is used. The variations with significant frequency difference in the cases and controls were subjected to further analysis.

### Scoring patients and normal individuals

The cumulative score of all variations in a patient is the Patient Score. All the variations in each patient were scored in all normal individuals and the average of this is the Normal Score. Patient and Normal Scores were estimated to predict involvement of mitochondrial variations in disease.

### Estimating performance of prediction

A cumulative frequency distribution curve is used to calculate the threshold score, to distinguish patients from normal subjects. Number of patients correctly picked, having score more than the threshold score are 'True positives' and patients that are wrongly classified, having score less than the threshold, is 'False negative'. Similarly, normal subjects, classified as patients, having a score more than threshold are 'False positives'. On the contrary, normal subjects correctly classified are 'True negatives'. Using these four attributes performance-estimating parameters, namely, accuracy (The percentage of predictions that are correct), sensitivity (The percentage of patients that were predicted as patients), specificity (The percentage of normal individuals that were predicted as normal), precision (The percentage of positive predictions that are correct), Kerr (The fraction of false predictions) and Matthews correlation coefficient (MCC) were calculated.

### Age of onset and disease severity

Our method has an optional age of onset parameter in the scoring scheme that can be used to assign scores if the impact of age needs to be evaluated. Selection of normal samples becomes significant in this aspect wherein data regarding the age of normal individuals should also be included in the analysis.

### The "And" logic for assessing cumulative impact of variations

The cumulative scores were obtained using the "AND" logic, where individual scores were multiplied. The AND logic implements logical conjunction meaning a high output results only if the inputs are high. The basic reason behind selecting "AND" logic was two fold. First, to evaluate the cumulative effect of variations and second, to account for the highly polymorphic nature of mtDNA, where variations neither reported nor predicted to have a role in disease are not considered even if they are informative based on their frequency difference from the control dataset. Thus, in absence of additional information for a variation, the score falls below threshold and mere presence of variation is not scored.

## List of abbreviations used

MtDNA: (mitochondrial DNA); mtSNP: (Japanese Mitochondrial Database); mtDB (Human Mitochondrial Genome Database).

## Competing interests

The authors declare that they have no competing interests.

## Authors' contributions

AB conceived and designed the study, wrote programs, performed analysis and drafted the manuscript. MM and ST participated in conceiving the study, provided inputs in design of the study and helped in reviewing the manuscript drafts. SS, JP, AKS, AB and MM were involved in acquisition and analysis of the Ataxia data. CSG was involved in developing the MtSNPscore web server along with AB. All authors read and approved the final manuscript.

## Supplementary Material

Additional file 1 **Variation selected from literature with details**. The table lists all the genes in mtDNA along with their OMIM identifiers. This is followed by phenotype/disease information obtained from OMIM/MitoMap/PubMed. As described in the text, each variation in assigned a Weighted Score, shown in brackets below the variation (Format – wild base Position mutated base (WS)). Number of reports is the number of published references considered for the variation and background mutations/modifiers are the mutations reported to modify their effect. This is followed by a brief description of the variation from the published reports and these reports are listed in the last column with links through PubMed identifiers. Click here for file

Additional file 2 **Distribution of variation reported in literature across mtDNA with score summary**. 2A. Gene-wise distribution of variation selected from published reports as described in text. 2B. Summary of scores assigned to these variations. Score of three, six and eight, were assigned to 24, 68 and 28 reported mutations, respectively. Click here for file

Additional file 3 **Summary of predictions of *in silico *tools for the reported variation**. Result of the reported variation in the protein coding genes analyzed by SIFT, PolyPhen, PhD-SNP and PLHOST. The mutation is reported along with disease and predictions made by the four *in silico *tools. The number in the brackets in case of PhD-SNP prediction indicates the Reliability Index for the prediction. The worksheet named "tRNA" lists published mutations missed by the evolution based computational analysis, most of which are in the loop region. Click here for file

Additional file 4 **Summary of predictions by *in silico *tools**. Summary of predictions of reported mutations. It can be seen that all the 43 reported mutations were predicted deleterious by at least one method, followed by 30 being predicted by two, 16 by three and two by all the four *in-silico *methods. Click here for file

Additional file 5 **Results obtained from Ataxia and mtSNP variation analysis**. AT – Ataxia; AD – Alzheimer's Disease; PD – Parkinson's Disease; T2DA – Type 2 Diabetes with Angiopathy; T2D – Type 2 Diabetes without Angiopathy; OB – Obese phenotype; TH – Thin phenotype; ID – Sample ID; Position – Position of mutation in the mitochondrial genome (as per rCRS); Locus – Gene name; Gene – Position of mutation in the gene; Prot – Position of mutation in the protein (In tRNAs, if mutation maps to loop or stem region (WC)); Nb – Normal base with frequency in normal sample set; Mb – Mutated base; Ncod – Normal codon; Mcod – Mutated codon; Nu – Normal codon usage; Mu – Mutated codon usage; Na – Normal amino acid; Ma – Mutated amino acid; Cod – Position of base change within the codon; ScoreP – Score assiged to mutated base in patients; Dloop – Variation mapping to regulatory important regions in Dloop; HSP – Heavy Strand Promoter; C I/III – Complex I/III reported mutation; 12SrRNA – 12SrRNA reported mutation; tRNA – tRNA reported mutation; Tol – Predicted tolerated by SIFT (T – Tolerated); Intol – Predicted 'Intolerant' by SIFT (I – Intolerant & I-LC – Intolerant with Low Confidence); Ben – Predicted 'Benign' by PolyPhen (B – Benign); Pos – Predicted 'Possibly Damaging' by PolyPhen (D – Possibly Damaging); Prob – Predicted 'Probably Damaging' by PolyPhen (D – Probably Damaging); PL – Predicted to change invariant peptides by PLHOST (in – Invariant); Del – Predicted Deleterious by Compensatory-co evolution method (Del – Deleterious); Phenotype – All the phenotypes where this mutation gets a high score; Conserved – Status of conservation of amino acid changes in various orders class Mammalia (obtained from mtDB). Click here for file

Additional file 6**Summary of performance estimating parameters**. Patient Max is the maximum score estimated for the patients in each disease category. AT – Ataxia; AD – Alzheimer's Disease; PD – Parkinson's Disease; T2DA – Type2 Diabetes With Angiopathy; T2D – Type2 Diabetes; OB – Obese; TH – Thin; MCC – Matthews correlation coefficient; TP – True Positive; TN – True Negative; FP – False Positive; FN – False Negative; Kerr – The fraction of false predictions.1. Accuracy (TN+TP)/(TP+TN+FP+FN)2. Precision TP/(TP+FP)3. Specificity TN/(TN+FP)4. Sensitivity TP/(TP+FN)5. MCC 6. Kerr (FP+FN)/TOTALClick here for file

Additional file 7 **Ataxia variation data**. Variation data from Ataxia patients. The sites (position of the change on the mtDNA) are reported as per rCRS. The involvement of trinucleotide repeats expansion in all the known ataxia genes was excluded in all these samples. Click here for file

Additional file 8 **Summary of overlapping bases in mtDNA**. The table enlists 12 overlapping genes in mtDNA along with their coordinates. Since scoring is done gene-wise for each individual these overlapping regions are scored twice, depending on the predictions/reported variations for the two genes sharing the overlapping base (s). Click here for file
