# Supplementary material for: MtSNPscore: a combined evidence approach for assessing cumulative impact of mitochondrial variations in disease
Source: BMC Bioinformatics. 2009 Aug 27;10(Suppl 8):S7. doi: 10.1186/1471-2105-10-S8-S7 (PMC2745589; doi:10.1186/1471-2105-10-S8-S7)
Supplement: Additional file 1 — Variation selected from literature with details. The table lists all the genes in mtDNA along with their OMIM identifiers. This is followed by phenotype/disease information obtained from OMIM/MitoMap/PubMed. As described in the text, each variation in assigned a Weighted Score, shown in brackets below the variation (Format – wild base Position mutated base (WS)). Number of reports is the number of published references considered for the variation and background mutations/modifiers are the mutations reported to modify their effect. This is followed by a brief description of the variation from the published reports and these reports are listed in the last column with links through PubMed identifiers. [file 1471-2105-10-S8-S7-S1.doc]

| **s.**  **no.** | **Gene name** | **OMIM ID** | **Phenotype/**  **disease** | **Variation**  **(Score assigned)** | **No. of reports** | **Background Mutations/**  **Modifiers** | **Description** | **References** |
| --- | --- | --- | --- | --- | --- | --- | --- | --- |
| **1** | **ND1** | 516000 | LHON  (mitochondrial complex i deficiency, included) | G3460A  (8) | >2 |  | Converts the modestly conserved alanine 52 to a threonine (A52T). It is sufficient by itself to cause LHON | [Brown et al., 1992](http://www.ncbi.nlm.nih.gov/entrez/query.fcgi?cmd=Retrieve&db=PubMed&dopt=Abstract&list_uids=1732158); [Howell et al., 1991](http://www.ncbi.nlm.nih.gov/entrez/query.fcgi?cmd=Retrieve&db=PubMed&dopt=Abstract&list_uids=1928099); [Howell et al., 1992](http://www.ncbi.nlm.nih.gov/entrez/query.fcgi?cmd=Retrieve&db=PubMed&dopt=Abstract&list_uids=1734726); [Huoponen et al., 1991](http://www.ncbi.nlm.nih.gov/entrez/query.fcgi?cmd=Retrieve&db=PubMed&dopt=Abstract&list_uids=1674640); [Johns, 1992](http://www.ncbi.nlm.nih.gov/entrez/query.fcgi?cmd=Retrieve&db=PubMed&dopt=Abstract&list_uids=1550131); [Johns et al., 1992](http://www.ncbi.nlm.nih.gov/entrez/query.fcgi?cmd=Retrieve&db=PubMed&dopt=Abstract&list_uids=1444915); [Majander et al., 1991](http://www.ncbi.nlm.nih.gov/entrez/query.fcgi?cmd=Retrieve&db=PubMed&dopt=Abstract&list_uids=1959619); [Paulus et al., 1993](http://www.ncbi.nlm.nih.gov/entrez/query.fcgi?cmd=Retrieve&db=PubMed&dopt=Abstract&list_uids=8496715); [Hinttala et al. (2006)](http://www.ncbi.nlm.nih.gov/entrez/query.fcgi?cmd=Retrieve&db=PubMed&dopt=Abstract&list_uids=16738010) |
|  |  |  |  |  |  |  | Created cybrids using a neuronal precursor cell line. LHON-NT2 neurons produced more superoxide than the undifferentiated controls supports the notion that the LHON mutations are causing a differentiation-dependent pathophysiological change, rather than, the simple inhibition of differentiation. | [Wong et al. (2002)](http://www.ncbi.nlm.nih.gov/entrez/query.fcgi?cmd=Retrieve&db=PubMed&dopt=Abstract&list_uids=11854175) |
|  |  |  | LHON | T3394C  (4) | >2 | mutation is rare among European LHON patients and found in about 0.9% of controls. It has only been observed on 1 haplotype and when combined with MTND6*LHON14484A is associated with blindness in 37% of maternal relatives, 100% of which are males | Converts the highly conserved tyrosine at amino acid 30 to a histidine (Y30H) | [Brown et al., 1992](http://www.ncbi.nlm.nih.gov/entrez/query.fcgi?cmd=Retrieve&db=PubMed&dopt=Abstract&list_uids=1732158); [Johns et al., 1992](http://www.ncbi.nlm.nih.gov/entrez/query.fcgi?cmd=Retrieve&db=PubMed&dopt=Abstract&list_uids=1444915); [Obayashi et al., 1992](http://www.ncbi.nlm.nih.gov/entrez/query.fcgi?cmd=Retrieve&db=PubMed&dopt=Abstract&list_uids=1442494) |
|  |  |  | LHON | G3733A  (6) | 1 |  | Glu143-to-lys (E143K) substitution in a conserved part of extra membrane loop facing the matrix side of the inner mt membrane.  Magnetic resonance spectroscopy disclosed an *in vivo* brain and skeletal muscle energy metabolism deficit in the four examined patients. Muscle biopsy from two patients showed slight mitochondrial proliferation with abnormal mitochondria. Biochemical investigations in platelets showed partially insensitive complex I to rotenone inhibition. | [Valentino et al. (2004)](http://www.ncbi.nlm.nih.gov/entrez/query.fcgi?cmd=Retrieve&db=PubMed&dopt=Abstract&list_uids=15505787) |
|  |  |  | MELAS | G3697A  (8) | 2 |  | Gly131-to-ser, deficiency of complex I activity in both skeletal muscle and cultured fibroblasts | [Kirby et al. (2004)](http://www.ncbi.nlm.nih.gov/entrez/query.fcgi?cmd=Retrieve&db=PubMed&dopt=Abstract&list_uids=15466014) |
|  |  |  |  | G3946A  (6) | 1 |  | 3946 perturb the critical structure in a conserved loop of the ND1 (glu214-to-lys). *Escherichia coli* and *Paracoccus denitrificans* as model systems to study the effect of this mutation. Expressed a specific deficiency of complex I activity in both skeletal muscle and cultured fibroblasts | [Marko Kervinen et al (2006)](http://hmg.oxfordjournals.org/cgi/content/abstract/15/17/2543)  [Kirby et al. (2004)](http://www.ncbi.nlm.nih.gov/entrez/query.fcgi?cmd=Retrieve&db=PubMed&dopt=Abstract&list_uids=15466014) |
|  |  |  |  | T3949C  (6) | 1 |  | 3949 perturb the critical structure in a conserved loop of the ND1(tyr215-to-his). *Escherichia coli* and *Paracoccus denitrificans* as model systems to study the effect of this mutation. Expressed a specific deficiency of complex I activity in both skeletal muscle and cultured fibroblasts. | [Kirby et al. (2004)](http://www.ncbi.nlm.nih.gov/entrez/query.fcgi?cmd=Retrieve&db=PubMed&dopt=Abstract&list_uids=15466014) |
|  |  |  | Leigh syndrome, to childhood MELAS, to adult-onset encephalopathic syndromes of variable severity | G3481A  (6) | 1 |  | Biochemically defined CI defect. Mutant transmitochondrial cybrids showed tight correlation between mutation load and decrease in CI activity, supporting the pathogenic role of the novel mutations. Structural studies on mutant cybrids showed impaired assembly or reduced stability of the holoenzyme complex. | [Zeviani M et al (2007)](http://www.ncbi.nlm.nih.gov/entrez/query.fcgi?cmd=Retrieve&db=PubMed&dopt=Abstract&list_uids=17535832) |
|  |  |  | Cardiomyopathy | G3337A  (4) | 1 | tRNALeu(CUN) A12308G and tRNAThr C15946T mutations | Mitochondrial respiratory chain complex I activity of the patients platelets was decreased compared to those of controls. Co-existence of mutations in tRNA and ND1 genes may act synergistically affecting the clinical phenotype | [Stathopoulos C.(2008)](http://www.ncbi.nlm.nih.gov/entrez/query.fcgi?cmd=Retrieve&db=PubMed&dopt=Abstract&list_uids=18502698) |
| **2** | **ND2** | 516001 | Complex I deficiency | G4810A  (6) | 1 |  | transition causing a change in tryptophan to a stop codon at position 114. This change predicted a truncated ND2 protein with a loss of 233 amino acids from the C terminus. The mutation was heteroplasmic in muscle and absent from blood. Single-fiber PCR analysis revealed a positive correlation between the proportion of mutant mtDNA and abnormal | [Pulkes et al. (2005)](http://www.ncbi.nlm.nih.gov/entrez/query.fcgi?cmd=Retrieve&db=PubMed&dopt=Abstract&list_uids=15781840) |
|  |  |  |  |  |  |  |  |  |
| **3** | **ND3** | 516002 | Complex I deficiency | T10191C  (8) | 2 |  | The mutation changes a hydrophilic serine in codon 45 to a hydrophobic proline (ser45 to pro; S45P), which may affect the folding of the protein. Mutation cause disproportionately greater reductions in enzyme activity than in the amount of fully assembled complex I | [Taylor et al. (2001](http://www.ncbi.nlm.nih.gov/entrez/query.fcgi?cmd=Retrieve&db=PubMed&dopt=Abstract&list_uids=11456298))  [McFarland et al. (2004)](http://www.ncbi.nlm.nih.gov/entrez/query.fcgi?cmd=Retrieve&db=PubMed&dopt=Abstract&list_uids=14705112) |
|  |  |  | Leigh syndrome due to mitochondrial complex I deficiency | T10158C  (8) | 2 |  | Iisolated complex I deficiency. increased blood and CSF lactate, delayed motor development, and bilateral thalamic lesions on MRI | [McFarland et al. (2004)](http://www.ncbi.nlm.nih.gov/entrez/query.fcgi?cmd=Retrieve&db=PubMed&dopt=Abstract&list_uids=14705112)  [Kirby et al. (2004)](http://www.ncbi.nlm.nih.gov/entrez/query.fcgi?cmd=Retrieve&db=PubMed&dopt=Abstract&list_uids=15466014) |
|  |  |  | Leigh syndrome (LS) or dystonia | G10197A  (6) | 2 | nuclear modifier genes may also play a role in the phenotypic expression and severity | Biochemical defect involving complex I and the discovery of the 10197G>A mutation with a similar phenotype in three unrelated families establish its pathogenicity and demonstrate that the amino acid position A47 is important for the function of complex I. Furthermore, this defect could be transferred along with the mutant mtDNAs to rho degrees lymphoblastoid cells in cybrid experiments | [Sarzi E et al (2007)](http://www.ncbi.nlm.nih.gov/entrez/query.fcgi?cmd=Retrieve&db=PubMed&dopt=Abstract&list_uids=17152068)  [Chae JH et al (2007)](http://www.ncbi.nlm.nih.gov/entrez/query.fcgi?cmd=Retrieve&db=PubMed&dopt=Abstract&list_uids=17413873) |
|  |  |  |  |  |  |  |  |  |
| **4** | **ND4** | 516003 | Leber optic atrophy | G11778A  (6) | >2 | 11778 and 14484 mutations showed a strong preferential association with haplotype J. The findings suggested that one ancient combination of haplotype J with specific mutations increases the penetrance of the 2 primary mutations 11778 and 14484.  Cybrid with background mutation 3640 | The allele changes the highly conserved arginine at amino acid 340 to a histidine (R340H).  This allele accounts for over 50% of Leber hereditary optic neuropathy (LHON; [535000](http://www.ncbi.nlm.nih.gov/entrez/dispomim.cgi?id=535000)) cases among Caucasians and over 90% of the cases in Asians  Cybrid cells containing the 11778G-A mutation showed a 60% reduction in the rate of complex I-dependent ATP synthesis compared to wildtype cells | [Wallace et al., 1988](http://www.ncbi.nlm.nih.gov/entrez/query.fcgi?cmd=Retrieve&db=PubMed&dopt=Abstract&list_uids=3201231)  [Singh et al., 1989](http://www.ncbi.nlm.nih.gov/entrez/query.fcgi?cmd=Retrieve&db=PubMed&dopt=Abstract&list_uids=2566116)  [Torroni et al. (1997)](http://www.ncbi.nlm.nih.gov/entrez/query.fcgi?cmd=Retrieve&db=PubMed&dopt=Abstract&list_uids=9150158) |
|  |  |  | Leigh syndrome,  Late-onset encephalopathy | C11777A  (8) | 2 |  | Cybrid study revealed that the percentage of heteroplasmy was correlated with complex I function and that the novel mutation caused a much more deleterious effect than the np 11,778 LHON mutation in complex I activity. | [Komaki H et al (2003)](http://www.ncbi.nlm.nih.gov/entrez/query.fcgi?cmd=Retrieve&db=PubMed&dopt=Abstract&list_uids=16120329)  [Deschauer M et al (2003)](http://www.ncbi.nlm.nih.gov/entrez/query.fcgi?cmd=Retrieve&db=PubMed&dopt=Abstract&list_uids=12707444) |
| **5** | **ND4L** | 516004 | Leber optic atrophy | T10663C  (4) | 1 | 10663C is a primary LHON mutation that is pathogenic when co-occurring with haplogroup J. | Analysis of complex I function in patient lymphoblasts and transmitochondrial cybrids revealed a partial complex I defect similar in magnitude to the 14484C mutation. | [Brown et al. (2002)](http://www.ncbi.nlm.nih.gov/entrez/query.fcgi?cmd=Retrieve&db=PubMed&dopt=Abstract&list_uids=11935318) |
| **6** | **ND5** | 516005 | Leigh syndrome due to mitochondrial complex I deficiency | T12706C  (4) | 1 |  | The mutation is predicted to change an invariant amino acid in a highly conserved trans membrane helix of the mature polypeptide & was hetero  plasmic. With a specific bio-  chemical defect involving complex I is highly suggestive of a pathogenic role for this mutation | [Taylor et al. (2002)](http://www.ncbi.nlm.nih.gov/entrez/query.fcgi?cmd=Retrieve&db=PubMed&dopt=Abstract&list_uids=11938446) |
|  |  |  | Melas syndrome included ,  Leigh syndrome due to mitochondrial complex I deficiency,  Leigh disease | G13513A  (8) | >2 |  | Mutation causes a complex I defect when present at unusually low mutant loads and may act dominantly. affects an evolutionarily conserved amino acid (D393N) | [Santorelli et al. (1997)](http://www.ncbi.nlm.nih.gov/entrez/query.fcgi?cmd=Retrieve&db=PubMed&dopt=Abstract&list_uids=9299505)  [Kirby et al. (2003)](http://www.ncbi.nlm.nih.gov/entrez/query.fcgi?cmd=Retrieve&db=PubMed&dopt=Abstract&list_uids=14520659)  [Chol et al. (2003)](http://www.ncbi.nlm.nih.gov/entrez/query.fcgi?cmd=Retrieve&db=PubMed&dopt=Abstract&list_uids=12624137)  [Sudo et al. (2004)](http://www.ncbi.nlm.nih.gov/entrez/query.fcgi?cmd=Retrieve&db=PubMed&dopt=Abstract&list_uids=14730434),  [Dickerson et al. (2005)](http://www.ncbi.nlm.nih.gov/entrez/query.fcgi?cmd=Retrieve&db=PubMed&dopt=Abstract&list_uids=16306525)  [Wong LJ (2008)](http://www.ncbi.nlm.nih.gov/entrez/query.fcgi?cmd=Retrieve&db=PubMed&dopt=Abstract&list_uids=18495510) |
|  |  |  | MELAS,  Leigh or Leigh-like encephalopathy, papillary thyroid carcinomas | A13514G  (4) | 2 |  | The 13514 A to G mutation resulted in a change of highly conserved aspartic acid to a low conserved glycine at amino acid position 393 and was not detected in the data of 119 normal controls with no thyroid cancer, 172 ethnically related patients with various mitochondrial diseases, or in ethnically similar Middle-eastern groups published previously at the Human Mitochondrial Genome Database | [Abu-Amero K et al (2004)](http://www.ncbi.nlm.nih.gov/entrez/query.fcgi?cmd=Retrieve&db=PubMed&dopt=Abstract&list_uids=15521990)  [Lebon S (2003)](http://www.ncbi.nlm.nih.gov/entrez/query.fcgi?cmd=Retrieve&db=PubMed&dopt=Abstract&list_uids=14684687) |
|  |  |  | Leigh syndrome, to childhood MELAS, to adult-onset encephalopathic syndromes of variable severity | G13063A  (6) | 1 |  | Biochemically defined CI defect. Mutant transmitochondrial cybrids showed tight correlation between mutation load and decrease in CI activity, supporting the pathogenic role of the novel mutations. Structural studies on mutant cybrids showed impaired assembly or reduced stability of the holoenzyme complex. | [Zeviani M et al (2007)](http://www.ncbi.nlm.nih.gov/entrez/query.fcgi?cmd=Retrieve&db=PubMed&dopt=Abstract&list_uids=17535832) |
|  |  |  |  |  |  |  |  |  |
| **7** | **ND6** | 516006 | Leber optic atrophy | T14484C  (4) | >2 | shown to occur on the haplogroup J background more frequently than expected. suggested a pathogenic role for a subset of LHON secondary mutations and their interaction with primary mutations | Mutation affects the highly conserved helix C, the interaction site of ubiquinol products | [Brown et al., 1992](http://www.ncbi.nlm.nih.gov/entrez/query.fcgi?cmd=Retrieve&db=PubMed&dopt=Abstract&list_uids=1732158);  [Carelli et al. (1999)](http://www.ncbi.nlm.nih.gov/entrez/query.fcgi?cmd=Retrieve&db=PubMed&dopt=Abstract&list_uids=10072046)  [Liu M et al., (2008)](http://www.ncbi.nlm.nih.gov/entrez/query.fcgi?cmd=Retrieve&db=PubMed&dopt=Abstract&list_uids=18440284) |
|  |  |  | Leber optic atrophy and dystonia, leigh syndrome due to mitochondrial complex I deficiency, included | G14459A  (6) | >2 |  | Broad spectrum of clinical manifestations due to complex I deficiency | [Gropman et al. (2004)](http://www.ncbi.nlm.nih.gov/entrez/query.fcgi?cmd=Retrieve&db=PubMed&dopt=Abstract&list_uids=14735585)  [Jun et al., 1994](http://www.ncbi.nlm.nih.gov/entrez/query.fcgi?cmd=Retrieve&db=PubMed&dopt=Abstract&list_uids=8016139);  [Wallace et al., 1994](http://www.ncbi.nlm.nih.gov/entrez/query.fcgi?cmd=Retrieve&db=PubMed&dopt=Abstract&list_uids=8016139),  [Kirby et al. (2000)](http://www.ncbi.nlm.nih.gov/entrez/query.fcgi?cmd=Retrieve&db=PubMed&dopt=Abstract&list_uids=10894222) |
|  |  |  | Leber optic atrophy and dystonia | T14596A  (6) | 1 |  | Biochemical analysis of a muscle biopsy showed severe complex I deficiency | [De Vries et al. (1996)](http://www.ncbi.nlm.nih.gov/entrez/query.fcgi?cmd=Retrieve&db=PubMed&dopt=Abstract&list_uids=8644732) |
|  |  |  | MELAS | G14453A  (4) | 1 | Identified 2 other homoplasmic mutations in the mtDNA of their patient, 5628T-C in the MTTA gene ([590000](http://www.ncbi.nlm.nih.gov/entrez/dispomim.cgi?id=590000)) and 13535A-G in the MTND5 gene ([516005](http://www.ncbi.nlm.nih.gov/entrez/dispomim.cgi?id=516005)), which might have contributed to the observed decrease in activity of complex I and the severe phenotype of the patient | Mitochondrial enzyme analysis in the patient showed a decreased activity of complex I in muscle | [Ravn et al.](http://www.ncbi.nlm.nih.gov/entrez/query.fcgi?cmd=Retrieve&db=PubMed&dopt=Abstract&list_uids=11781695)(2001) |
|  |  |  | Leigh syndrome due to mitochondrial complex I deficiency, striatal necrosis, bilateral, with dystonia, included | T14487C  (8) | >2 |  | Mutation occurred in the most conserved transmembrane helix of the protein and caused an impaired assembly of complex I. Analysis showed isolated complex I deficiency | [Ugalde et al. (2003)](http://www.ncbi.nlm.nih.gov/entrez/query.fcgi?cmd=Retrieve&db=PubMed&dopt=Abstract&list_uids=14595656)  [Solano et al. (2003)](http://www.ncbi.nlm.nih.gov/entrez/query.fcgi?cmd=Retrieve&db=PubMed&dopt=Abstract&list_uids=14520668)  [Funalot et al., 2002](http://www.ncbi.nlm.nih.gov/entrez/query.fcgi?cmd=Retrieve&db=PubMed&dopt=Abstract&list_uids=12205655)) |
|  |  |  |  |  |  |  |  |  |
|  |  |  | LHON | C14482G  (4) | 2 | found on a haplogroup J mtDNA background | In any case, the M64I amino acid substitution was associated with a good prognosis in both the Italian and Turkish families, suggesting that at least this feature is not modulated by the mtDNA haplogroup | [Valentino ML (2002)](http://www.ncbi.nlm.nih.gov/entrez/query.fcgi?cmd=Retrieve&db=PubMed&dopt=Abstract&list_uids=12112086) |
|  |  |  | LHON | C14568T  (4) | 2 |  | Lie within the evolutionarily most conserved region of the ND6 gene in a hydrophobic pocket making it a hot spot for the disease | [Fauser S et al (2002)](http://www.ncbi.nlm.nih.gov/entrez/query.fcgi?cmd=Retrieve&db=PubMed&dopt=Abstract&list_uids=12324878) |
|  |  |  | Leigh syndrome, to childhood MELAS, to adult-onset encephalopathic syndromes of variable severity | G14600A  (6) | 1 |  | Biochemically defined CI defect. Mutant transmitochondrial cybrids showed tight correlation between mutation load and decrease in CI activity, supporting the pathogenic role of the novel mutations. Structural studies on mutant cybrids showed impaired assembly or reduced stability of the holoenzyme complex. | [Zeviani M et al (2007)](http://www.ncbi.nlm.nih.gov/entrez/query.fcgi?cmd=Retrieve&db=PubMed&dopt=Abstract&list_uids=17535832) |
|  |  |  |  |  |  |  |  |  |
| **8** | **CYTB** | 516020 | Leber optic atrophy | G15257A  (4) | >2 | In most, but not all cases, the MTCYB*LHON15257A allele has been associated with the primary LHON mutation MTND6*LHON14484A and the secondary mutation MTND5*LHON13708A. Individuals harboring this mtDNA haplotype may also harbor the allele MTCYB*LHON15812A, and the MTCYB*LHON15257A allele has been found in association with the MTND2*LHON5244A mutation in 1 case | Displays characteristics of both primary and secondary LHON mutations and hence may significantly contribute to the disease process | [Brown et al., 1992](http://www.ncbi.nlm.nih.gov/entrez/query.fcgi?cmd=Retrieve&db=PubMed&dopt=Abstract&list_uids=1732158);  [Huoponen et al., 1993](http://www.ncbi.nlm.nih.gov/entrez/query.fcgi?cmd=Retrieve&db=PubMed&dopt=Abstract&list_uids=7901141); [Johns and Neufeld, 1991](http://www.ncbi.nlm.nih.gov/entrez/query.fcgi?cmd=Retrieve&db=PubMed&dopt=Abstract&list_uids=1764087) |
|  |  |  | Leber optic atrophy | G15812A  (4) | 2 | This is a secondary LHON mutation which is regularly found associated with the additional LHON mutations: MTND5*LHON13708A, MTND6*LHON14484A, and MTCYB*LHON15257A | While the mutation does not cause LHON, it may increase the probability of phenotypic expression | [Brown et al., 1992](http://www.ncbi.nlm.nih.gov/entrez/query.fcgi?cmd=Retrieve&db=PubMed&dopt=Abstract&list_uids=1732158);  [Johns and Neufeld, 1991](http://www.ncbi.nlm.nih.gov/entrez/query.fcgi?cmd=Retrieve&db=PubMed&dopt=Abstract&list_uids=1764087) |
|  |  |  | Exercise intolerance | G15615A  (8) | >2 |  | Reviewed in certain cases, low activity of respiratory chain complex III in muscle | [Dumoulin et al. (1996)](http://www.ncbi.nlm.nih.gov/entrez/query.fcgi?cmd=Retrieve&db=PubMed&dopt=Abstract&list_uids=8910895)  [Andreu et al. (1999)](http://www.ncbi.nlm.nih.gov/entrez/query.fcgi?cmd=Retrieve&db=PubMed&dopt=Abstract&list_uids=10502593) |
|  |  |  |  |  |  |  |  |  |
|  |  |  | Exercise intolerance | G14846A  (6) | 1 |  | The patient showed 'ragged-red fibers' (22%) on muscle biopsy. The nucleotide substitution resulted in a gly34-to-ser amino acid substitution + Lactic acidosis | [Andreu et al. (1999)](http://www.ncbi.nlm.nih.gov/entrez/query.fcgi?cmd=Retrieve&db=PubMed&dopt=Abstract&list_uids=10502593) |
|  |  |  | Encephalomyopathy | G15242A  (6) | 1 |  | Gly166-to-ter truncation of the protein with loss of 215 amino acids representing 57% of the C terminus of cytochrome b | [Keightley et al. (2000)](http://www.ncbi.nlm.nih.gov/entrez/query.fcgi?cmd=Retrieve&db=PubMed&dopt=Abstract&list_uids=11047755) |
|  |  |  | Exercise intolerance | G15150A  (6) | 1 |  | Trp135-to-ter truncation of the protein. complex III activity was extremely decreased, Western blot analysis of complex III in muscle showed undetectable cytochrome b protein | [Legros et al. (2001)](http://www.ncbi.nlm.nih.gov/entrez/query.fcgi?cmd=Retrieve&db=PubMed&dopt=Abstract&list_uids=11464242) |
|  |  |  | Exercise intolerance | T15197C  (6) | 1 |  | Ser151-to-pro amino acid substitution. The serine at position 151 was highly conserved and the mutation constituted an important modification in the physicochemical properties of the amino acid. complex III activity was extremely decreased, Western blot analysis of complex III in muscle showed a reduced amount of cytochrome b protein | [Legros et al. (2001)](http://www.ncbi.nlm.nih.gov/entrez/query.fcgi?cmd=Retrieve&db=PubMed&dopt=Abstract&list_uids=11464242) |
|  |  |  | Multisystem disorder | A15579G  (6) | 1 |  | Decrease of muscle complex III activity. changed a highly conserved tyrosine to cysteine | [Wibrand et al. (2001)](http://www.ncbi.nlm.nih.gov/entrez/query.fcgi?cmd=Retrieve&db=PubMed&dopt=Abstract&list_uids=11601507) |
|  |  |  | Cardiomyopathy, infantile histiocytoid | G15498A  (6) | 1 |  | Thought to impair the function of the holoenzyme as deduced from its effects on the crystal structure of ubiquinol cytochrome c oxidoreductase | [Andreu et al. (2000)](http://www.ncbi.nlm.nih.gov/entrez/query.fcgi?cmd=Retrieve&db=PubMed&dopt=Abstract&list_uids=10960495) |
|  |  |  | Parkinsonism/MELAS overlap Syndrome | 4-BP DEL, 14787TTAA  (6) | 1 |  | This mutation in this highly conserved gene is considered to be pathogenic since it is a heteroplasmic frame shift mutation predicted to lead to a truncated protein. | [De Coo et al. (1999)](http://www.ncbi.nlm.nih.gov/entrez/query.fcgi?cmd=Retrieve&db=PubMed&dopt=Abstract&list_uids=9894888) |
| **9** | **COX1** | 516030 | Leber optic atrophy, leber hereditary optic neuropathy; lhon deafness, aminoglycoside-induced, included deafness, nonsyndromic sensorineural, included | G7444A  (6) | >2 | Patients with this mutation cluster within the Caucasian mtDNA phylogenetic tree. In 2 cases studied also harbor other LHON mutations: the ND1*LHON3460A mutation in one case and the ND6*LHON14484A in the other. This mutations is probably a secondary LHON mutation.  Unrelated Mongolian deaf students with cosegregation of a G7444A mutation and a A1555G mutation in the MTRNR1 gene.  G7444A mutation share a common pathogenic mechanism as the adjacent 7445A-G mutationin the MTTS1 gene, which results in aberrant processing of the tRNA-ser(UCN) precursor | Converts the AGA termination codon to a lysine codon (AAA), permitting extension of the MTCO1 polypeptide by 3 amino acids (lysine, glutamine, lysine) into the antisense tRNAser(UCN) sequence. Mutation is associated with an impaired mobility of the polypeptide on SDS-PAGE and a 36% reduction in Complex IV activity in patient lymphoblasts | [Brown et al., 1992](http://www.ncbi.nlm.nih.gov/entrez/query.fcgi?cmd=Retrieve&db=PubMed&dopt=Abstract&list_uids=1732158); [Johns and Neufield, 1993](http://www.ncbi.nlm.nih.gov/entrez/query.fcgi?cmd=Retrieve&db=PubMed&dopt=Abstract&list_uids=8240356),  [Brown and Wallace, 1994](http://www.ncbi.nlm.nih.gov/entrez/query.fcgi?cmd=Retrieve&db=PubMed&dopt=Abstract&list_uids=8077181), [Pandya et al. (1999)](http://www.ncbi.nlm.nih.gov/entrez/query.fcgi?cmd=Retrieve&db=PubMed&dopt=Abstract&list_uids=10577941),  [Yuan et al. (2005),](http://www.ncbi.nlm.nih.gov/entrez/query.fcgi?cmd=Retrieve&db=PubMed&dopt=Abstract&list_uids=16152638)  [Guan et al., 1998](http://www.ncbi.nlm.nih.gov/entrez/query.fcgi?cmd=Retrieve&db=PubMed&dopt=Abstract&list_uids=9742104) |
|  |  |  | Cytochrome c oxidase deficiency | G6930A  (6) | 1 |  | glycine codon to a stop codon, resulting in the predicted loss of the last 170 amino acids (33%) of the polypeptide.  The genetic, biochemical, and morphologic characteristics of trans-mt cybrid cell lines, obtained by fusing platelets from the patient with human cells lacking endogenous mtDNA. There was a direct relationship between the proportion of mutant mtDNA and the biochemical defect. They also observed that the threshold for the phenotypic expression of this mutation was lower than that reported in mutations involving tRNA genes. It is suggested that this mutation causes a disruption in the assembly of the respiratory-chain complex IV | [Bruno et al. (1999)](http://www.ncbi.nlm.nih.gov/entrez/query.fcgi?cmd=Retrieve&db=PubMed&dopt=Abstract&list_uids=10441567) |
|  |  |  | Cytochrome c oxidase I deficiency | C6328T  (6) | 1 |  | Ser142-to-phe (S142F) substitution in the beginning of the fourth N-terminal transmembrane helix. Expression of the homologous mutation in the bacterium *Paracoccus denitrificans* resulted in a significant decrease in COX enzyme activity. | [Lucioli et al. (2006)](http://www.ncbi.nlm.nih.gov/entrez/query.fcgi?cmd=Retrieve&db=PubMed&dopt=Abstract&list_uids=16284789) |
|  |  |  | Cytochrome c oxidase I deficiency | C6489A  (4) | 1 |  | The point mutation led to a substitution of ile at the highly conserved leu196 (L196I). Muscle biopsy showed in single fibers decreased COX activity and lowered binding of COX antibodies, suggesting decreased stability of the mutated enzyme. | [Varlamov et al. (2002)](http://www.ncbi.nlm.nih.gov/entrez/query.fcgi?cmd=Retrieve&db=PubMed&dopt=Abstract&list_uids=12140182) |
| **10** | **COX2** | 516040 | Cytochrome c oxidase deficiency | T7671A  (6) | 1 |  | A marked reduction in COX activity, mutation changed a methionine to a lysine residue in the middle of the first N-terminal membrane-spanning region of COX II, a structural association of COX II with COX I is necessary to stabilize the binding of heme a3 to COX I | [Rahman et al. (1999)](http://www.ncbi.nlm.nih.gov/entrez/query.fcgi?cmd=Retrieve&db=PubMed&dopt=Abstract&list_uids=10486321) |
|  |  |  | Cytochrome c oxidase deficiency | T7587C  (4) | 1 |  | Muscle biopsy samples revealed isolated COX deficiency and mitochondrial proliferation. | [Clark et al. (1999)](http://www.ncbi.nlm.nih.gov/entrez/query.fcgi?cmd=Retrieve&db=PubMed&dopt=Abstract&list_uids=10205264) |
|  |  |  | Cytochrome c oxidase deficiency | G7896A  (6) | 1 |  | Nonsense mutation, predicted to cause premature termination of the translation, with loss of 123 amino acids at the C terminus of COX II | [Campos et al. (2001)](http://www.ncbi.nlm.nih.gov/entrez/query.fcgi?cmd=Retrieve&db=PubMed&dopt=Abstract&list_uids=11558799) |
| **11** | **COX3** | 516050 | Cytochrome c oxidase deficiency | 15-BP DEL  (6) | 1 |  | Muscle histochemistry -many ragged-red fibers stained heavily for succinate dehydrogenase (SDH) activity, and a high proportion (64%) of COX-negative fibers. On electron microscopy, increased numbers of mitochondria, some enlarged and irregular, with disordered cristae. Amino acid loss in a highly conserved region, included 2 highly conserved Phe residues. Immunoblots and immunocytochemistry suggested a lack of assembly or instability of the complex. | [Keightley et al. (1996)](http://www.ncbi.nlm.nih.gov/entrez/query.fcgi?cmd=Retrieve&db=PubMed&dopt=Abstract&list_uids=8630495) |
|  |  |  | Cytochrome c oxidase deficiency | G9952A  (6) | 1 |  | Mutation is located in the 3-prime end of the gene and predicted to result in the loss of the last 13 amino acids of the highly conserved C-terminal region of this subunit. Biochemical studies confirmed a severe isolated reduction in COX activity. Muscle immunocytochemistry revealed a pattern suggestive of a primary mtDNA defect in the COX-deficient fibers and was consistent with either reduced stability or impaired assembly of the holoenzyme | [Hanna et al. (1998)](http://www.ncbi.nlm.nih.gov/entrez/query.fcgi?cmd=Retrieve&db=PubMed&dopt=Abstract&list_uids=9634511) |
|  |  |  | Cytochrome c oxidase deficiency | G9379A  (4) | 1 |  | Immunohistochemistry showed a decreased steady state level of COX subunits II and III in skeletal muscle. trp58-to-ter mutation. | [Horvath et al. (2002)](http://www.ncbi.nlm.nih.gov/entrez/query.fcgi?cmd=Retrieve&db=PubMed&dopt=Abstract&list_uids=12414820) |
|  |  |  | Cytochrome c oxidase deficiency | 1-BP INS, 9537C  (6) | 1 |  | No full-length COX subunit III protein was detected in mtDNA translation assays *in vivo*.  The fully assembled complex was absent. The authors proposed a role for COX subunit III in the incorporation and maintenance of smaller COX subunits within the complex | [Tiranti et al. (2000)](http://www.ncbi.nlm.nih.gov/entrez/query.fcgi?cmd=Retrieve&db=PubMed&dopt=Abstract&list_uids=11063732) |
|  |  |  |  |  |  |  |  |  |
| **12** | **ATP6** | 516060 | Leigh syndrome narp syndrome, included | T8993G  (8) | >2 |  | Mutation induces a structural defect in F1F0-ATPase that causes a severe impairment of ATP synthesis.  Mitochondrial transformant cybrids. Immunoblot analysis revealed an abnormal amount of subcomplexes, F1-ATPase and V.The cybrids had decreased subcomplex V assembly and decreased ATP synthesis capacity. However, the cells had no marked phenotype, suggesting that the effects of this mutation are subtle and have no effect on cell viability.  Allotopic expression of stably transfected constructs in cybrids homoplasmic with respect to the 8993T-G mutation showed a significantly improved recovery after growth in selective medium as well as a significant increase in ATP synthesis | [Harding et al. (1992)](http://www.ncbi.nlm.nih.gov/entrez/query.fcgi?cmd=Retrieve&db=PubMed&dopt=Abstract&list_uids=1539598), [Holt et al. (1990)](http://www.ncbi.nlm.nih.gov/entrez/query.fcgi?cmd=Retrieve&db=PubMed&dopt=Abstract&list_uids=2137962), [Tatuch et al. (1992)](http://www.ncbi.nlm.nih.gov/entrez/query.fcgi?cmd=Retrieve&db=PubMed&dopt=Abstract&list_uids=1550128), [Shoffner et al. (1992)](http://www.ncbi.nlm.nih.gov/entrez/query.fcgi?cmd=Retrieve&db=PubMed&dopt=Abstract&list_uids=1436530), [Nijtmans et al. (2001)](http://www.ncbi.nlm.nih.gov/entrez/query.fcgi?cmd=Retrieve&db=PubMed&dopt=Abstract&list_uids=11076946), [Manfredi et al. (2002)](http://www.ncbi.nlm.nih.gov/entrez/query.fcgi?cmd=Retrieve&db=PubMed&dopt=Abstract&list_uids=11925565) |
|  |  |  | Leigh syndrome ataxia and polyneuropathy, adult-onset, included | T8993C  (6) | >2 |  | Reported in various case studies with different disorders. | [van Erven et al., 1987](http://www.ncbi.nlm.nih.gov/entrez/query.fcgi?cmd=Retrieve&db=PubMed&dopt=Abstract&list_uids=3612192), [de Vries et al. (1993)](http://www.ncbi.nlm.nih.gov/entrez/query.fcgi?cmd=Retrieve&db=PubMed&dopt=Abstract&list_uids=8395787), [Chakrapani et al. (1998)](http://www.ncbi.nlm.nih.gov/entrez/query.fcgi?cmd=Retrieve&db=PubMed&dopt=Abstract&list_uids=9762610), [Fujii et al. (1998),](http://www.ncbi.nlm.nih.gov/entrez/query.fcgi?cmd=Retrieve&db=PubMed&dopt=Abstract&list_uids=9568930) [Vilarinho et al. (2001)](http://www.ncbi.nlm.nih.gov/entrez/query.fcgi?cmd=Retrieve&db=PubMed&dopt=Abstract&list_uids=11916326), [Rantamaki et al. (2005)](http://www.ncbi.nlm.nih.gov/entrez/query.fcgi?cmd=Retrieve&db=PubMed&dopt=Abstract&list_uids=16049925) |
|  |  |  | Bilateral striatal necrosis, infantile, leigh syndrome | T9176C  (6) | >2 |  | Changed the highly conserved leucine to proline | [Thyagarajan et al. (1995)](http://www.ncbi.nlm.nih.gov/entrez/query.fcgi?cmd=Retrieve&db=PubMed&dopt=Abstract&list_uids=7668837), [Dionisi-Vici et al. (1998)](http://www.ncbi.nlm.nih.gov/entrez/query.fcgi?cmd=Retrieve&db=PubMed&dopt=Abstract&list_uids=9501263),  [Makino et al. (1998)](http://www.ncbi.nlm.nih.gov/entrez/query.fcgi?cmd=Retrieve&db=PubMed&dopt=Abstract&list_uids=9631394) |
| **13** | **ATP8** | 516070 | Cardiomyopathy, Apical Hypertrophic And Neuropathy | G8529A  (6) | 1 |  | Reduced complex V activity was measured in the patient's fibroblasts and muscle tissue, and was confirmed in cybrid clones containing patient-derived mitochondrial DNA. | [Jonckheere et al. (2008)](http://www.ncbi.nlm.nih.gov/entrez/query.fcgi?cmd=Retrieve&db=PubMed&dopt=Abstract&list_uids=17954552) |
|  |  |  |  |  |  |  |  |  |
| **14** | **12SrRNA** | 561000 | Deafness, aminoglycoside-induced,  Deafness, nonsyndromic, senso-rineural,  Cardiomyopathy | A1555G  (6) | >2 | mtDNA backgrounds probably do not play a major role in disease expression | Mutation was inferred to create a new basepair at the terminus of the penultimate helix of the 12S RNA. [Hutchin et al. (1993)](javascript:Anchor('561000_Reference20')) proposed that this additional basepair decreases the molecular volume taken up by RNA at this site relative to the unpaired bases, thus increasing the size of the aminoglycoside binding pocket and making aminoglycoside binding tighter. Predicted to change the RNA secondary structure.  As per [Young et al. (2005)](javascript:Anchor('561000_Reference36')) and [Dai et al. (2006)](javascript:Anchor('561000_Reference11')), mutation itself is not sufficient to produce the clinical phenotype | [Pandya et al. (1997)](http://www.ncbi.nlm.nih.gov/entrez/query.fcgi?cmd=Retrieve&db=PubMed&dopt=Abstract&list_uids=9039999), [Gardner et al. (1997)](http://www.ncbi.nlm.nih.gov/entrez/query.fcgi?cmd=Retrieve&db=PubMed&dopt=Abstract&list_uids=9391883), [Estivill et al. (1998)](http://www.ncbi.nlm.nih.gov/entrez/query.fcgi?cmd=Retrieve&db=PubMed&dopt=Abstract&list_uids=9490575), [Santorelli et al. (1999)](http://www.ncbi.nlm.nih.gov/entrez/query.fcgi?cmd=Retrieve&db=PubMed&dopt=Abstract&list_uids=9915970).  [Young et al. (2005)](http://www.ncbi.nlm.nih.gov/entrez/query.fcgi?cmd=Retrieve&db=PubMed&dopt=Abstract&list_uids=15708009) and [Dai et al. (2006)](http://www.ncbi.nlm.nih.gov/entrez/query.fcgi?cmd=Retrieve&db=PubMed&dopt=Abstract&list_uids=16375862), |
|  |  |  | Deafness, aminoglycoside-induced deafness, sensorineural, included auditory neuropathy | T1095C  (6) | >2 |  | Predicted effect of the mutation is to destroy the stem-loop secondary structure, resulting in impaired translation. Significant decrease of COX activity.  [Yao et al. (2006)](javascript:Anchor('561000_Reference35')) concluded that the mutation defines a basal haplotype branch of the East Asian mtDNA phylogeny, and is thus likely non-pathogenic. The authors also presented evidence disputing the pathogenicity of other reported mutations in the 12S rRNA gene associated with hearing loss | [Thyagarajan et al. (2000)](http://www.ncbi.nlm.nih.gov/entrez/query.fcgi?cmd=Retrieve&db=PubMed&dopt=Abstract&list_uids=11079536), [Tessa et al. (2001)](http://www.ncbi.nlm.nih.gov/entrez/query.fcgi?cmd=Retrieve&db=PubMed&dopt=Abstract&list_uids=11313749),  [Yao et al. (2006)](http://www.ncbi.nlm.nih.gov/entrez/query.fcgi?cmd=Retrieve&db=PubMed&dopt=Abstract&list_uids=16528519) |
|  |  |  | Deafness, Nonsyndromic Sensorineural | T1291C  (4) | 1 |  | mutation is predicted to change the RNA secondary structure | [Ballana et al. (2006)](http://www.ncbi.nlm.nih.gov/entrez/query.fcgi?cmd=Retrieve&db=PubMed&dopt=Abstract&list_uids=16458854) |
| **15** | **16SrRNA** | 561010 |  |  |  |  |  |  |
|  |  |  |  |  |  |  |  |  |
| **16** | **tRNA-Ala** | 590000 | Myotonic Dystrophy-Like Myopathy | G5650A  (6) | 1 |  | Several cytochrome c oxidase-negative ragged-red fibers together with clinically observed exercise intolerance and lactic acidosis pointed to a mitochondrial origin. Single-fiber PCR showed a significantly higher rate of mutant mtDNA in ragged-red fibers | [Horvath et al. (2003)](http://www.ncbi.nlm.nih.gov/entrez/query.fcgi?cmd=Retrieve&db=PubMed&dopt=Abstract&list_uids=14569122) |
|  |  |  | Mitochondrial Myopathy | G5591A  (6) | 1 |  | Muscle biopsy showed ragged red fibers and cytochrome c oxidase deficiency, and PCR analysis of muscle tissue detected 98% mutant mtDNA | [Swalwell et al. (2006)](http://www.ncbi.nlm.nih.gov/entrez/query.fcgi?cmd=Retrieve&db=PubMed&dopt=Abstract&list_uids=16476954) |
| **17** | **tRNA-Arg** | 500005 | Encephalomyopathy | A10438G  (4) | 1 |  | Defect in the mitochondrial respiratory chain and abnormal ultrastructure of muscle mitochondria suggests a clinically probable mitochondrial encephalopathy | [Uusimaa et al. (2004)](http://www.ncbi.nlm.nih.gov/entrez/query.fcgi?cmd=Retrieve&db=PubMed&dopt=Abstract&list_uids=15286228) |
|  |  |  |  |  |  |  |  |  |
| **18** | **tRNA-Asn** | 590010 | Isolated ophthalmoplegia | G5703A  (8) | >2 |  | The mutation disrupted the first basepair of the anticodon stem, a secondary structure highly conserved throughout evolution.  Gel electrophoresis showed that the mutant tRNA fraction had an altered conformation consistent with a destabilized secondary or tertiary structure, which may result in impaired aminoacylation or increased *in vivo* tRNA degradation by mitochondrial RNases | [Moraes et al. (1993)](http://www.ncbi.nlm.nih.gov/entrez/query.fcgi?cmd=Retrieve&db=PubMed&dopt=Abstract&list_uids=8254046), [Hao and Moraes (1997)](http://www.ncbi.nlm.nih.gov/entrez/query.fcgi?cmd=Retrieve&db=PubMed&dopt=Abstract&list_uids=9372914),  [Vives-Bauza et al. (2003)](http://www.ncbi.nlm.nih.gov/entrez/query.fcgi?cmd=Retrieve&db=PubMed&dopt=Abstract&list_uids=14518831) |
|  |  |  | Ophthalmoplegia, Isolated | A5692G  (4) | 1 |  | The mutation is located at the transition of the anticodon loop to the anticodon stem and reduces the number of loop-forming nucleotides from 7 to 5, predicting a limitation in mitochondrial protein synthesis. | [Seibel et al. (1994)](http://www.ncbi.nlm.nih.gov/entrez/query.fcgi?cmd=Retrieve&db=PubMed&dopt=Abstract&list_uids=7980504) |
|  |  |  | Mitochondrial Complex I Deficiency | A5728G  (6) | 1 |  | Muscle tissue and cultured skin fibroblasts show a combined deficiency of complexes I and IV. An identical biochemical profile was seen in transmitochondrial cybrids carrying more than 55% mutant mitochondrial DNA | [Meulemans et al. (2006)](http://www.ncbi.nlm.nih.gov/entrez/query.fcgi?cmd=Retrieve&db=PubMed&dopt=Abstract&list_uids=16908752) |
| **19** | **tRNA-Asp** | 590015 | Mitochondrial Myopathy, Isolated | A7526G  (6) | 1 |  | Decreased complex I and IV enzyme activity and ragged red fibers on muscle biopsy | [Seneca et al. (2005)](http://www.ncbi.nlm.nih.gov/entrez/query.fcgi?cmd=Retrieve&db=PubMed&dopt=Abstract&list_uids=16059939) |
|  |  |  |  |  |  |  |  |  |
| **20** | **tRNA-Cys** | 590020 | Oculopharyngeal somatic myopathy,  Mitochondrial neurogastrointestinal encephalomyopathy (MNGIE) | T5814C  (6) | 2 | large-scale 3,399 base pair (bp) deletion of the mitochondrial DNA (mtDNA).  In MNGIE, mutations also reported in the thymidine phosphorylase gene (ECGF1). | Biopsy showed decreased enzymatic activity of cytochrome c oxidase, but no ragged-red fibers. Electron microscopy showed "parking-lot" paracrystalline inclusions in the enlarged mitochondria suggestive for mitochondrial myopathy. | [Thajeb P et al (2006)](http://www.ncbi.nlm.nih.gov/entrez/query.fcgi?cmd=Retrieve&db=PubMed&dopt=Abstract&list_uids=16644408)  [Carod-Artal FJ et al (2007)](http://www.ncbi.nlm.nih.gov/entrez/query.fcgi?cmd=Retrieve&db=PubMed&dopt=Abstract&list_uids=17437622) |
|  |  |  |  |  |  |  |  |  |
| **21** | **tRNA-Glu** | 590025 | Myopathy, mitochondrial, with diabetes mellitus  Diabetes-deafness syndrome, maternally transmitted | T14709C  (4) | >2 | Nuclear factors expected to be involved in expression of the pathogenic effect of this mutation | Altered an evolutionarily conserved nucleotide in the region specifying for the anticodon loop of mitochondrial tRNA.  [Perucca-Lostanlen et al. (2002)](http://www.ncbi.nlm.nih.gov/entrez/query.fcgi?cmd=Retrieve&db=PubMed&dopt=Abstract&list_uids=12393175) suggested that additional nuclear factors may be involved in expression of the phenotype | [Hao et al. (1995)](http://www.ncbi.nlm.nih.gov/entrez/query.fcgi?cmd=Retrieve&db=PubMed&dopt=Abstract&list_uids=7726154), [McFarland et al. (2004)](http://www.ncbi.nlm.nih.gov/entrez/query.fcgi?cmd=Retrieve&db=PubMed&dopt=Abstract&list_uids=15048886), [Vialettes et al. (1997)](http://www.ncbi.nlm.nih.gov/entrez/query.fcgi?cmd=Retrieve&db=PubMed&dopt=Abstract&list_uids=9353617).  [Perucca-Lostanlen et al. (2002)](http://www.ncbi.nlm.nih.gov/entrez/query.fcgi?cmd=Retrieve&db=PubMed&dopt=Abstract&list_uids=12393175) |
|  |  |  |  |  |  |  |  |  |
| **22** | **tRNA-Gln** | 590030 | Myopathy | 1-BP INS, 4366A  (6) | 1 |  | Altered the length of the evolutionarily conserved anticodon loop from 7 to 8 bases.  A muscle biopsy of the patient showed ragged-red fibers and an unusually high percentage of cytochrome c oxidase-deficient fibers (89%) | [Dey et al. (2000)](http://www.ncbi.nlm.nih.gov/entrez/query.fcgi?cmd=Retrieve&db=PubMed&dopt=Abstract&list_uids=10996779) |
|  |  |  | MELAS | G4332A  (8) | 2 |  | The pathogenicity of the mutation was shown in single muscle fibers by the correlation between high mutation load and cytochrome c oxidase defect | [M. Bataillard et al (2001)](http://www.ncbi.nlm.nih.gov/entrez/query.fcgi?cmd=Retrieve&db=PubMed&dopt=Abstract&list_uids=11171912) |
|  |  |  |  |  |  |  |  |  |
| **23** | **tRNA-Gly** | 590035 | Cardiomyopathy, hypertrophic | T9997C  (6) | 1 |  | The mutation disrupted hydrogen bonding in the region adjacent to the acceptor stem of the tRNA molecule, highly conserved in mammals, as well as in various vertebrates and invertebrates.  The degree of heteroplasmy in lymphoblast cultures also correlated with the level of enzyme activity present in complex IV and complexes II and III.  Electron microscopy identified pleomorphic mitochondria with abnormal cristae. | [Merante et al. (1994)](http://www.ncbi.nlm.nih.gov/entrez/query.fcgi?cmd=Retrieve&db=PubMed&dopt=Abstract&list_uids=8079988) |
|  |  |  | Exercise intolerance | T10010C  (6) | 1 |  | Increased serum lactic acid, and intermittent elevated creatine kinase. Histochemical and biochemical analysis of muscle tissue revealed COX-negative ragged-red fibers (RRF) and COX-negative non-RRF, as well as reduced activities of mitochondrial respiratory chain complexes I, III, and IV | [Nishigaki et al. (2002)](http://www.ncbi.nlm.nih.gov/entrez/query.fcgi?cmd=Retrieve&db=PubMed&dopt=Abstract&list_uids=11971101) |
|  |  |  | sudden infant death syndrome | A10044G  (8) | 2 |  | Mutation inhibits *in vitro* CCA addition to the tRNA by the human mitochondrial CCA-adding enzyme. The mutant tRNA(gly) showed high nuclease sensitivity in both the T and the D loops, suggesting a weakened interaction between the loops | [Tomari et al. (2003)](http://www.ncbi.nlm.nih.gov/entrez/query.fcgi?cmd=Retrieve&db=PubMed&dopt=Abstract&list_uids=12621050),  [Santorelli et al. (1996)](http://www.ncbi.nlm.nih.gov/entrez/query.fcgi?cmd=Retrieve&db=PubMed&dopt=Abstract&list_uids=8888049) |
|  |  |  |  |  |  |  |  |  |
| **24** | **tRNA-His** | 590040 | Cardiomyopathy, idiopathic dilated, mitochondrial, LHON | G12192A  (4) | 2 | G11778A ND4 gene | Mutation was located 2 bp from the 3-prime end of the T-psi-C loop of the tRNA. Because the mutation added an A:T basepair and shortened the loop itself, it was thought to affect mitochondrial function | [Shin et al. (2000)](http://www.ncbi.nlm.nih.gov/entrez/query.fcgi?cmd=Retrieve&db=PubMed&dopt=Abstract&list_uids=11038324), [Mimaki et al. (2003)](http://www.ncbi.nlm.nih.gov/entrez/query.fcgi?cmd=Retrieve&db=PubMed&dopt=Abstract&list_uids=12560876) |
|  |  |  |  |  |  | A16318T in D-loop |  |  |
|  |  |  | MERRF/MELAS overlap syndrome | G12147A  (8) | 2 |  | Muscle biopsy of the proband showed abnormal mitochondrial proliferation and COX-negative fibers with decreased respiratory chain function | [Melone et al. (2004)](http://www.ncbi.nlm.nih.gov/entrez/query.fcgi?cmd=Retrieve&db=PubMed&dopt=Abstract&list_uids=14967777),  [Taylor et al. (2004)](http://www.ncbi.nlm.nih.gov/entrez/query.fcgi?cmd=Retrieve&db=PubMed&dopt=Abstract&list_uids=15111688) |
|  |  |  | Pigmentary Retinopathy and Sensorineural Deafness | G12183A  (4) | 1 |  | The mutation is in a highly conserved region of the T(psi)C stem of the tRNA(His) gene and may alter secondary structure formation. | [Crimi et al. (2003)](http://www.ncbi.nlm.nih.gov/entrez/query.fcgi?cmd=Retrieve&db=PubMed&dopt=Abstract&list_uids=12682337) |
| **25** | **tRNA-Ile** | 590045 | Fatal infantile cardiomyopathy | A4317G  (8) | 2 |  | Mutation inhibits *in vitro* CCA addition to the tRNA by the human mitochondrial CCA-adding enzyme. They observed a structural rearrangement of the T-arm region, conferring an aberrantly stable T-arm structure and an increased T(m) value. | [Tanaka et al. (1990)](http://www.ncbi.nlm.nih.gov/entrez/query.fcgi?cmd=Retrieve&db=PubMed&dopt=Abstract&list_uids=1978914),  [Tomari et al. (2003)](http://www.ncbi.nlm.nih.gov/entrez/query.fcgi?cmd=Retrieve&db=PubMed&dopt=Abstract&list_uids=12621050) |
|  |  |  | Cardiomyo fatal pathy | A4269G  (6) | 1 |  | Histopathologic findings included mitochondrial myopathy with ragged-red fibers and focal cytochrome c oxidase-deficient fibers in skeletal and cardiac muscles | [Taniike et al. (1992)](http://www.ncbi.nlm.nih.gov/entrez/query.fcgi?cmd=Retrieve&db=PubMed&dopt=Abstract&list_uids=1632786) |
|  |  |  | Chronic progressive external ophthalmoplegia and multiple sclerosis | G4298A  (8) | 2 |  | Mutation alters an evolutionary conserved nucleotide within the anticodon stem, heteroplasmic in skeletal muscle but was not present in the patient's blood. Single fibre PCR analysis revealed significantly higher levels of the G4298A mutation in cytochrome c oxidase (COX) negative fibres than in COX-positive fibres. | [Taylor RW et al (1998)](http://www.ncbi.nlm.nih.gov/entrez/query.fcgi?cmd=Retrieve&db=PubMed&dopt=Abstract&list_uids=9473477) |
|  |  |  | Maternally inherited hypertrophic cardiomyopathy | A4300G  (6) | 2 | coexistence of mtDNA mutations in patients with beta myosin heavy chain (beta MHC) linked hypertrophic cardiomyopathy (HCM) | Cardiac tissue from an affected child in the presenting family exhibited severe deficiencies of mitochondrial respiratory chain enzymes, whereas histochemical and biochemical studies of the skeletal muscle were normal. The pathogenic role for this mutation was confirmed by high-resolution Northern blot analysis of heart tissue from both families, revealing very low steady-state levels of the mature mitochondrial tRNA(Ile). | [Taylor RW et al (2003)](http://www.ncbi.nlm.nih.gov/entrez/query.fcgi?cmd=Retrieve&db=PubMed&dopt=Abstract&list_uids=12767666)  [Arbustini E et al (1998)](http://www.ncbi.nlm.nih.gov/entrez/query.fcgi?cmd=Retrieve&db=PubMed&dopt=Abstract&list_uids=10065021) |
|  |  |  | Hypertension, hypercholesterolemia, and hypomagnesemia, mitochondrial | T4291C  (4) | 1 |  | Homoplasmic mutation substituting cytidine for uridine immediately 5' to the mt tRNA(Ile) anticodon. Uridine at this position is nearly invariate among transfer RNAs because of its role in stabilizing the anticodon loop. Given the known loss of mitochondrial function with aging, these findings may have implications for the common clustering of these metabolic disorders. | [Wilson et al. (2004)](http://www.ncbi.nlm.nih.gov/entrez/query.fcgi?cmd=Retrieve&db=PubMed&dopt=Abstract&list_uids=15498972) |
| **26** | **tRNA-Leu**  **(UUR)** | 590050 | Melas syndrome, diabetes-deafness syndrome, maternally transmitted,  muscle stiffness, painful, 3-@methylglutaconic aciduria, maculopathy, age-related, cyclic vomiting syndrome | A3243G  (6) | >2 | A11084G mutation of ND4.  [Torroni et al. (2003)](javascript:Anchor('590050_Reference63')) concluded that the 3243A-G mutation may harbor all the evolutionary features expected from a severely deleterious mtDNA mutation under strong negative selection, and they reveal that European mtDNA backgrounds do not play a substantial role in modulating the mutation's phenotypic expression | By histochemical, immuno-histochemical, & single-fiber PCR analysis, [Moraes et al. (1992)](javascript:Anchor('590050_Reference40')) demonstrated that ragged-red fibers were associated with high levels of mutant mitochondrial genomes and with partial cytochrome c oxidase deficiency.  Mutation lies within a DNA segment responsible for transcription termination of the rRNA genes.  3243 mutation occurs independently in the maternal lineages of most MELAS patients.  [Lam et al. (1997)](javascript:Anchor('590050_Reference30')) supported the suggestion that valproate should not be given to patients suspected of having mitochondrial diseases.  [Janssen et al. (1999)](javascript:Anchor('590050_Reference25')) showed that cells harboring patient-derived mitochondria A3243Gdisplay severe loss of respiration. Despite the low level of leucyl-tRNA-Leu(UUR), the rate of mitochondrial translation was not seriously affected. Therefore, it is not the decrease of protein synthesis but the mitochondrially encoded proteins seemed subject to elevated degradation, leading to a severe reduction in their steady state level, leading to a disequilibrium between the levels of mitochondrial and nuclear encoded respiratory chain subunits and thereby a reduction of functional respiratory chain complexes. [Chomyn et al. (2000)](javascript:Anchor('590050_Reference6')) presented several lines of evidence indicating that the protein synthesis defect in 3243A-G MELAS mutation-carrying cells is mainly due to a reduced association of mRNA with ribosomes, possibly as a consequence of the tRNA-leu(UUR) aminoacylation defect. | [Goto et al. (1990)](http://www.ncbi.nlm.nih.gov/entrez/query.fcgi?cmd=Retrieve&db=PubMed&dopt=Abstract&list_uids=2102678), [Kobayashi et al. (1990)](http://www.ncbi.nlm.nih.gov/entrez/query.fcgi?cmd=Retrieve&db=PubMed&dopt=Abstract&list_uids=2268345),  [Moraes et al. (1992)](http://www.ncbi.nlm.nih.gov/entrez/query.fcgi?cmd=Retrieve&db=PubMed&dopt=Abstract&list_uids=1315123),  [Lertrit et al. (1992)](http://www.ncbi.nlm.nih.gov/entrez/query.fcgi?cmd=Retrieve&db=PubMed&dopt=Abstract&list_uids=1323207),  [Morten et al. (1995)](http://www.ncbi.nlm.nih.gov/entrez/query.fcgi?cmd=Retrieve&db=PubMed&dopt=Abstract&list_uids=8541865),  [Lam et al. (1997)](http://www.ncbi.nlm.nih.gov/entrez/query.fcgi?cmd=Retrieve&db=PubMed&dopt=Abstract&list_uids=9243242),  [Janssen et al. (1999)](http://www.ncbi.nlm.nih.gov/entrez/query.fcgi?cmd=Retrieve&db=PubMed&dopt=Abstract&list_uids=10514449),  [Chomyn et al. (2000)](http://www.ncbi.nlm.nih.gov/entrez/query.fcgi?cmd=Retrieve&db=PubMed&dopt=Abstract&list_uids=10858457),  [Torroni et al. (2003)](http://www.ncbi.nlm.nih.gov/entrez/query.fcgi?cmd=Retrieve&db=PubMed&dopt=Abstract&list_uids=12612863),  [Pyle et al. (2007](http://www.ncbi.nlm.nih.gov/entrez/query.fcgi?cmd=Retrieve&db=PubMed&dopt=Abstract&list_uids=16950816)) |
|  |  |  | Melas | T3271C  (8) | 2 |  | Cybrid clones induced both low complex I activity and abnormal mtDNA-encoded polypeptide synthesis that included at least subunit ND6 of complex I | [Hayashi et al. (1993)](http://www.ncbi.nlm.nih.gov/entrez/query.fcgi?cmd=Retrieve&db=PubMed&dopt=Abstract&list_uids=8280119),  [Stenqvist et al. (2005)](http://www.ncbi.nlm.nih.gov/entrez/query.fcgi?cmd=Retrieve&db=PubMed&dopt=Abstract&list_uids=16006433) |
|  |  |  | Merrf, diabetes mellitus, noninsulin-dependent, maternally transmitted | C3256T  (8) | 2 |  | The enzyme activity of the mitochondrial oxidative phosphorylation pathway in the muscle of the proband was decreased, especially in complex I. Analysis of single muscle fibers indicated that morphologic and biochemical alterations appeared only when the proportion of mutant mtDNA exceeded 90% of the total cellular mtDNA pool | [Moraes et al. (1993)](http://www.ncbi.nlm.nih.gov/entrez/query.fcgi?cmd=Retrieve&db=PubMed&dopt=Abstract&list_uids=8254046),  [Hirai et al. (1998)](http://www.ncbi.nlm.nih.gov/entrez/query.fcgi?cmd=Retrieve&db=PubMed&dopt=Abstract&list_uids=9506761) |
|  |  |  | Cardiomyopathy with or without skeletal myopathy | C3303T  (6) | 2 |  | Mutation disrupted a conserved basepair in the aminoacyl stem of the tRNA-leu (UUR). Causative role of the C3303T mutation was confirmed by [Bruno et al. (1999)](javascript:Anchor('590050_Reference3')), who found the mutation in 8 patients from 4 unrelated families | [Silvestri et al. (1994)](http://www.ncbi.nlm.nih.gov/entrez/query.fcgi?cmd=Retrieve&db=PubMed&dopt=Abstract&list_uids=7906985),  [Bruno et al. (1999)](http://www.ncbi.nlm.nih.gov/entrez/query.fcgi?cmd=Retrieve&db=PubMed&dopt=Abstract&list_uids=10431114) |
|  |  |  | Mt Encephalomyopathy | T3252C  (6) | 1 |  | Muscle biopsy demonstrated 3%-10% ragged red fibers. 3252 is a highly conserved position. | [Morten et al. (1993)](http://www.ncbi.nlm.nih.gov/entrez/query.fcgi?cmd=Retrieve&db=PubMed&dopt=Abstract&list_uids=8111377) |
|  |  |  | Progressive external ophthalmoplegia, proximal myopathy, and sudden death | A3251G  (8) | 2 |  | Studies of isolated muscle mitochondria revealed a profound isolated complex I deficiency | [Sweeney et al. (1993)](http://www.ncbi.nlm.nih.gov/entrez/query.fcgi?cmd=Retrieve&db=PubMed&dopt=Abstract&list_uids=8265770),  [Houshmand et al. (1996)](http://www.ncbi.nlm.nih.gov/entrez/query.fcgi?cmd=Retrieve&db=PubMed&dopt=Abstract&list_uids=8786060) |
|  |  |  | Cardiomyopathy with or without skeletal myopathy | A3260G  (8) | 2 |  | Heart failure & muscle weakness due to skeletal myopathy with ragged-red fibers. From study of respiratory capacity and mitochondrial protein synthesis in transformant cybrids harboring the mutation, [Mariotti et al. (1994)](javascript:Anchor('590050_Reference35')) proved that the G3260 mutation was responsible for the clinical disorder in this family. | [Mariotti et al. (1994)](http://www.ncbi.nlm.nih.gov/entrez/query.fcgi?cmd=Retrieve&db=PubMed&dopt=Abstract&list_uids=8132749),  [Zeviani et al. (1991)](http://www.ncbi.nlm.nih.gov/entrez/query.fcgi?cmd=Retrieve&db=PubMed&dopt=Abstract&list_uids=1677065) |
|  |  |  | Skeletal myopathy, responsive to riboflavin | T3250C  (8) | 2 |  | Complex I deficiency and skeletal myopathy. | [Ogle et al. (1997)](http://www.ncbi.nlm.nih.gov/entrez/query.fcgi?cmd=Retrieve&db=PubMed&dopt=Abstract&list_uids=9003864),  [Goto et al. (1992)](http://www.ncbi.nlm.nih.gov/entrez/query.fcgi?cmd=Retrieve&db=PubMed&dopt=Abstract&list_uids=1514779) |
|  |  |  | Neuropsychiatric disorder and early-onset cataract | A3274G  (6) | 1 |  | Severe deficiency of respiratory complex I in skeletal muscle. Changes on brain MRI were progressive. | [Jaksch et al. (2001)](http://www.ncbi.nlm.nih.gov/entrez/query.fcgi?cmd=Retrieve&db=PubMed&dopt=Abstract&list_uids=11723298) |
|  |  |  | Kearns-sayre syndrome | G3249A  (6) | 1 |  | Skeletal muscle analysis showed decreased activity of complex I and numerous ragged-red fibers | [Seneca et al. (2001)](http://www.ncbi.nlm.nih.gov/entrez/query.fcgi?cmd=Retrieve&db=PubMed&dopt=Abstract&list_uids=11448301) |
|  |  |  | Myelodysplastic syndrome, somatic | G3242A  (6) | 1 |  | Pathologic iron accumulation in the mitochondria of erythroblasts.  mtDNA mutation in CD34+ cells was associated with a maturation defect. This effect contributed to ineffective hematopoiesis in the patient | [Gattermann et al. (2004)](http://www.ncbi.nlm.nih.gov/entrez/query.fcgi?cmd=Retrieve&db=PubMed&dopt=Abstract&list_uids=14576046) |
|  |  |  | MELAS | T3291C  (8) | >2 |  | T3291C mutants, which were the least charged by LeuRS, have fragile structures. In addition, the T3291C mutant can inhibit aminoacylation of the wild-type hmtRNALeu(UUR), indicating that it may act as an inhibitor in the mitochondrial heteroplasmic environment.  Yeast counterparts of this mutation were defective for growth on respiratory substrates, exhibited an abnormal mitochondrial morphology, and accumulated mitochondrial DNA deletions at a very high rate | [Hao R et al (2004)](http://www.ncbi.nlm.nih.gov/entrez/query.fcgi?cmd=Retrieve&db=PubMed&dopt=Abstract&list_uids=15581630)  [Feuermann M et al (2003)](http://www.ncbi.nlm.nih.gov/entrez/query.fcgi?cmd=Retrieve&db=PubMed&dopt=Abstract&list_uids=12524521) |
|  |  |  | Progressive mitochondrial myopathy with proximal muscle weakness | A3302G  (6) | 2 | Pathogenesis may be elicited through cumulative effects of tRNA mutations.  A high mutation load of the A3302G mutation can lead to fatal cardiorespiratory failure, likely triggered by low environmental oxygen pressure and exercise | Causes respiratory chain complex I deficiency. Pathogenesis is explained by abnormal mtRNA processing (reduced the efficiency of 3'-end cleavage). | [Hutchison WM et al (2005)](http://www.ncbi.nlm.nih.gov/entrez/query.fcgi?cmd=Retrieve&db=PubMed&dopt=Abstract&list_uids=16344351)  [Levinger L et al (2004)](http://www.ncbi.nlm.nih.gov/entrez/query.fcgi?cmd=Retrieve&db=PubMed&dopt=Abstract&list_uids=15019775) |
|  |  |  |  |  |  |  |  |  |
| **27** | **tRNA-Leu2**  **(CUN)** | 590055 | Mt Encephalo-myopathy | G12315A  (6) | 1 |  | Mutation disrupted basepairing in the T-psi-C stem of tRNAleu. Patient with ragged-red muscle fibers. | [Fu et al. (1996)](http://www.ncbi.nlm.nih.gov/entrez/query.fcgi?cmd=Retrieve&db=PubMed&dopt=Abstract&list_uids=8923013) |
|  |  |  | Myopathy, mitochondrial | A12320G  (6) | 1 |  | Mutation affected the T-psi-C loop at a conserved site. Skeletal muscle contained the mutation and demonstrated a biochemical defect of respiratory chain activity. In a series of 4 muscle biopsy specimens taken over a 12-year period, there was a gradual increase, from 70 to 90%, in the overall level of the mutation, as well as a marked clinical deterioration. Single-fiber PCR confirmed that the proportion of mutant mtDNA was highest in cytochrome c oxidase-negative fibers. | [Weber et al. (1997)](http://www.ncbi.nlm.nih.gov/entrez/query.fcgi?cmd=Retrieve&db=PubMed&dopt=Abstract&list_uids=9012410) |
|  |  |  | Cardiomyopathy, mitochondrial | T12297C  (6) | 1 |  | An endomyocardial biopsy showed wide variation in size and shape of the mitochondria, with several composed of peripheral rings of mitochondrial membranes and devoid of internal cristae. The mutation affected a highly conserved nucleotide. | [Grasso et al. (2001)](http://www.ncbi.nlm.nih.gov/entrez/query.fcgi?cmd=Retrieve&db=PubMed&dopt=Abstract&list_uids=11313776),  [Tessa et al., 1999](http://www.ncbi.nlm.nih.gov/entrez/query.fcgi?cmd=Retrieve&db=PubMed&dopt=Abstract&list_uids=10602359) |
|  |  |  |  |  |  |  |  |  |
| **28** | **tRNA-Lys** | 590060 | MERRF | A8344G  (6) | >2 | ATP6 gene (T8993G, T8993C) | Myoclonus epilepsy and ragged-red fibers.  Associated with a decrease in the activity of cytochrome c oxidase. Proteins of the complex I and VI subunits were more affected than complex V subunits, and there was a rough correlation with both protein size and number of lysine residues.  Functionally a recessive mutation that can be rescued by intraorganellar complementation.  Severe protein synthesis impairment in MERRF mutation-carrying cells was due to premature termination of translation at each or near each lysine codon, with the deficiency of aminoacylated tRNA-lys being the most likely cause of this phenomenon. [Borner et al. (2000)](javascript:Anchor('590060_Reference2')) reported that A8344G mutation does not affect tRNALys function in MERRF patients. [Chomyn et al. (1991)](javascript:Anchor('590060_Reference6')) showed thattRNA mutation itself is sufficient to cause the disease using cybrid assays. | [Shoffner et al. (1990)](http://www.ncbi.nlm.nih.gov/entrez/query.fcgi?cmd=Retrieve&db=PubMed&dopt=Abstract&list_uids=2112427)  [Yoneda et al. (1990)](http://www.ncbi.nlm.nih.gov/entrez/query.fcgi?cmd=Retrieve&db=PubMed&dopt=Abstract&list_uids=2124116),  [Boulet et al. (1992)](http://www.ncbi.nlm.nih.gov/entrez/query.fcgi?cmd=Retrieve&db=PubMed&dopt=Abstract&list_uids=1334369),  [Fang et al. (1994)](http://www.ncbi.nlm.nih.gov/entrez/query.fcgi?cmd=Retrieve&db=PubMed&dopt=Abstract&list_uids=8264702),  [Enriquez et al. (1995)](http://www.ncbi.nlm.nih.gov/entrez/query.fcgi?cmd=Retrieve&db=PubMed&dopt=Abstract&list_uids=7647790),  [Borner et al. (2000)](http://www.ncbi.nlm.nih.gov/entrez/query.fcgi?cmd=Retrieve&db=PubMed&dopt=Abstract&list_uids=10699170),  [Chomyn et al. (1991)](http://www.ncbi.nlm.nih.gov/entrez/query.fcgi?cmd=Retrieve&db=PubMed&dopt=Abstract&list_uids=1848674) |
|  |  |  | MERRF/MELAS overlap syndrome | T8356C  (8) | 2 |  | a highly conserved basepair in the T-psi-C stem disrupted. Muscle biopsy shows abundant ragged-red fibers. Amount of mutant mtDNA in muscle correlated withseverity of the clinical presentation | [Silvestri et al. (1992)](http://www.ncbi.nlm.nih.gov/entrez/query.fcgi?cmd=Retrieve&db=PubMed&dopt=Abstract&list_uids=1361099),  [Zeviani et al. (1993)](http://www.ncbi.nlm.nih.gov/entrez/query.fcgi?cmd=Retrieve&db=PubMed&dopt=Abstract&list_uids=8069654) |
|  |  |  | Cardiomyopathy and deafness | G8363A  (6) | 1 |  | Muscle biopsies from the probands showed mitochondrial proliferation and partial defects of complexes I, III, and IV of the electron-transport chain. | [Santorelli et al. (1996)](http://www.ncbi.nlm.nih.gov/entrez/query.fcgi?cmd=Retrieve&db=PubMed&dopt=Abstract&list_uids=8651277) |
|  |  |  | Myoneural gastrointestinal encephalopathy syndrome MNGIE | G8313A  (6) | 1 |  | Evolutionarily conserved nucleotide. Muscle biopsy revealed ragged-red fibers lacking cytochrome c oxidase activity and diminished levels of respiratory chain enzyme complexes. | [Verma et al. (1997)](http://www.ncbi.nlm.nih.gov/entrez/query.fcgi?cmd=Retrieve&db=PubMed&dopt=Abstract&list_uids=9380435) |
|  |  |  | MERRF | G8361A  (6) | 1 |  | Disrupted conserved base pairing interaction in the aminoacyl-acceptor stem of the encoded tRNA-lys. Maternally inherited, heteroplasmic in all tissues tested, and correlated with mitochondrial dysfunction in individual muscle fibers. | [Rossmanith et al. (2003)](http://www.ncbi.nlm.nih.gov/entrez/query.fcgi?cmd=Retrieve&db=PubMed&dopt=Abstract&list_uids=14681892) |
| **29** | **tRNA-Met** | 590065 | Splenic lymphoma | G4450A  (6) | 1 | although the pathogenicity of the mutation was clearly demonstrated, its link with the patient disease remained disputable | The pathogenic potential of the mutation was clearly established by the following criteria. It was absent in a control population. It involves a nucleotide that is highly conserved along the phylogenetic tree. The mutation was heteroplasmic and, when present in a high proportion, was associated with morphological alterations of the mitochondria, with defects of respiratory chain complexes activities and with a decrease in the mitochondrially encoded cytochrome c oxidase subunit II. Transfer of the mutation in Rho0 cells allowed to demonstrate its association with a severe respiratory chain dysfunction | [Lombes A et al (1998)](http://www.ncbi.nlm.nih.gov/entrez/query.fcgi?cmd=Retrieve&db=PubMed&dopt=Abstract&list_uids=9452079) |
|  |  |  | Mitochondrial myopathy | T4409C  (6) | 1 |  | This mutation has disrupted a critical Mg(2+)-binding site on the tRNA required for formation of the biologically active structure. | [Spremulli LL. (2008)](http://www.ncbi.nlm.nih.gov/entrez/query.fcgi?cmd=Retrieve&db=PubMed&dopt=Abstract&list_uids=18835817) |
| **30** | **tRNA-Phe** | 590070 | MELAS | G583A  (8) | >2 |  | Lactic acidemia, and ragged-red fibers.  G583 nucleotide is highly conserved. | [Shapira et al. (1975)](http://www.ncbi.nlm.nih.gov/entrez/query.fcgi?cmd=Retrieve&db=PubMed&dopt=Abstract&list_uids=1171391),  [Hanna et al. (1998)](http://www.ncbi.nlm.nih.gov/entrez/query.fcgi?cmd=Retrieve&db=PubMed&dopt=Abstract&list_uids=9771776) |
|  |  |  | Myopathy, mitochondrial, late-onset | G622A  (6) | 1 |  | Skeletal muscle biopsy detected atrophic muscle fibers, and histochemical analysis showed over 35% COX-negative fibers. Enzyme assay showed decreased activity of several respiratory chain complexes. | [Deschauer et al. (2006)](http://www.ncbi.nlm.nih.gov/entrez/query.fcgi?cmd=Retrieve&db=PubMed&dopt=Abstract&list_uids=16769874) |
|  |  |  |  |  |  |  |  |  |
| **31** | **tRNA-Pro** | 590075 | MYOPATHY | G15990A  (6) | 1 |  | Anticodon changed from UGG (tRNA-Pro) to UGA (tRNASer). Analysis of single muscle fibers indicated that mutant mtDNAs severely impaired mitochondrial protein synthesis and respiratory chain activity, but only when present at greater than 90%. The recessive behavior of this mtDNA alteration was thought to explain the patient's relatively mild clinical phenotype | [Moraes et al. (1993)](http://www.ncbi.nlm.nih.gov/entrez/query.fcgi?cmd=Retrieve&db=PubMed&dopt=Abstract&list_uids=7689388) |
|  |  |  | Parkinson disease, susceptibility | T15965C  (4) | 1 |  | This mutation alters position 64 of the TpsiC stem. The corresponding nucleotide in bacterial aminoacyl-tRNAs is involved in the interaction with elongation factor Tu. | [Grasbon-Frodl et al. (1999)](http://www.ncbi.nlm.nih.gov/entrez/query.fcgi?cmd=Retrieve&db=PubMed&dopt=Abstract&list_uids=10369889) |
| **32** | **tRNA-Ser** | 590080 | MERRF/MELAS overlap syndrome, cytochrome c oxidase deficiency | T7512C  (8) | 2 |  | Mutation disrupted a highly conserved basepair in the acceptor stem. mitochondrial encephalopathy and cytochrome c oxidase deficiency. (Explanation same as G7497A) | [Nakamura et al. (1995)](http://www.ncbi.nlm.nih.gov/entrez/query.fcgi?cmd=Retrieve&db=PubMed&dopt=Abstract&list_uids=7669057),  [Jaksch et al. (1998)](http://www.ncbi.nlm.nih.gov/entrez/query.fcgi?cmd=Retrieve&db=PubMed&dopt=Abstract&list_uids=9832034) |
|  |  |  | Keratoderma, palmoplantar, with deafness, Deafness, nonsyndromic sensorineural | A7445G  (6) | >2 | The penetrance and expressivity varied considerably among individuals, suggesting that additional environmental and/or genetic factors were involved. | The mutation caused an average reduction of approximately 70% in the level of tRNA-ser(UCN) and a decrease of approximately 45% in protein synthesis rate in the cell lines analyzed. Associated with the 7445A-G mutation was a marked reduction in the level of the mRNA for the ND6 subunit gene ([516006](http://www.ncbi.nlm.nih.gov/entrez/dispomim.cgi?id=516006)), which is located approximately 7 kb upstream and is cotranscribed with the tRNA-ser(UCN) gene. | [Guan et al. (1998)](http://www.ncbi.nlm.nih.gov/entrez/query.fcgi?cmd=Retrieve&db=PubMed&dopt=Abstract&list_uids=9742104),  [Reid et al. (1994)](http://www.ncbi.nlm.nih.gov/entrez/query.fcgi?cmd=Retrieve&db=PubMed&dopt=Abstract&list_uids=8019558),  [Fischel-Ghodsian et al. (1995)](http://www.ncbi.nlm.nih.gov/entrez/query.fcgi?cmd=Retrieve&db=PubMed&dopt=Abstract&list_uids=8572257)  [Hutchin et al. (2001)](http://www.ncbi.nlm.nih.gov/entrez/query.fcgi?cmd=Retrieve&db=PubMed&dopt=Abstract&list_uids=11175301) |
|  |  |  | Exercise intolerance, Myoclonus epilepsy and deafness | G7497A  (8) | >2 |  | Used osteosarcoma cybrids and reported severe reduction of tRNA(Ser(UCN)) to levels below 10% of controls for both mutations, resulting in a 40% reduction in mitochondrial protein synthesis rate and in a respiratory chain deficiency resembling that in the patients muscle. Aminoacylation was apparently unaffected. On non-denaturating northern blots we detected an altered electrophoretic mobility for G7497A containing tRNA molecules suggesting a structural impact of this mutation, which was confirmed by structural probing | [Mollers M et al (2005)](http://www.ncbi.nlm.nih.gov/entrez/query.fcgi?cmd=Retrieve&db=PubMed&dopt=Abstract&list_uids=16199753) |
|  |  |  | Nonsyndromic hearing loss (HL) | T7511C  (6) | >2 | Other modifiers and mitochondrial mutations (T3308C & T5655C) may modulate the penetrance of this mutation | The most significant histopathologic finding was severe loss of spiral ganglion cells in all turns of the cochlea. Severe loss of neuronal filaments in Rosenthal's canal was also observed. The organ of Corti showed scattered loss of inner and outer hair cells in the basal turn. Partial atrophy of the stria vascularis was observed in all turns of the cochlea.  Three cybrids derived from an affected matrilineal relative carrying the homoplasmic T7511C mutation, exhibited approximately 75% decrease in the tRNA(Ser(UCN)) level, compared with three control cybrids. This defect is likely a primary contributor to approximately 52% reduction in the rate of mitochondrial protein synthesis and marked defects in respiration and growth properties.  A muscle biopsy specimen from the proband showed cytochrome c oxidase (COX)-deficient fibers but no ragged-red fibers; biochemical analysis showed marked reduction of COX activity. Single-fiber PCR showed that the mutational load was greater in COX-deficient muscle fibers. | [Ishikawa K et al (2006)](http://www.ncbi.nlm.nih.gov/entrez/query.fcgi?cmd=Retrieve&db=PubMed&dopt=Abstract&list_uids=17075421)  [Li R et al (2005)](http://www.ncbi.nlm.nih.gov/entrez/query.fcgi?cmd=Retrieve&db=PubMed&dopt=Abstract&list_uids=15670746)  [Li X et al (2004)](http://www.ncbi.nlm.nih.gov/entrez/query.fcgi?cmd=Retrieve&db=PubMed&dopt=Abstract&list_uids=14960712)  [Sue CM et al (1999)](http://www.ncbi.nlm.nih.gov/entrez/query.fcgi?cmd=Retrieve&db=PubMed&dopt=Abstract&list_uids=10371545) |
|  |  |  | cytochrome c oxidase deficiency | 1-BP INS, 7472C  (6) | >2 |  | This mutation alone is sufficient to cause hearing loss and that when present at very high levels can also lead to neurologic dysfunction | [Hutchin et al. (2001)](http://www.ncbi.nlm.nih.gov/entrez/query.fcgi?cmd=Retrieve&db=PubMed&dopt=Abstract&list_uids=11378827)  [Friedman et al. (1999)](http://www.ncbi.nlm.nih.gov/entrez/query.fcgi?cmd=Retrieve&db=PubMed&dopt=Abstract&list_uids=10340654)  [Verhoeven et al. (1999)](http://www.ncbi.nlm.nih.gov/entrez/query.fcgi?cmd=Retrieve&db=PubMed&dopt=Abstract&list_uids=10094190) |
|  |  |  | Deafness, Nonsyndromic Sensorineural | T7510C  (4) | 1 |  | The mutation is predicted to disrupt basepairing in the acceptor stem of the tRNA | [Hutchin et al. (2001)](http://www.ncbi.nlm.nih.gov/entrez/query.fcgi?cmd=Retrieve&db=PubMed&dopt=Abstract&list_uids=10978361) |
| **33** | **tRNA-Ser2** | 590085 | Cerebellar ataxia, cataract, and diabetes mellitus, Retinitis pigmentosa-deafness syndrome, included | C12258A  (8) | 2 |  | In the acceptor stem, expected to effect both secondary and tertiary structure as well as the function of this tRNA. The mutated DNA was at much higher levels in the COX-negative compared with the COX-positive fibers. Mutation causes the observed biochemical defect in the muscle and presumably in the pancreatic beta cells, since insulin secretion is heavily dependent on oxidative metabolism, and mitochondrial dysfunction will impair insulin secretion. The consensus C at this position is highly conserved and is present in species as divergent from *Homo sapiens* as vulture and platypus. The mutation was thought to disrupt the amino acid-receptor stem of the tRNA molecule, affecting aminoacylation of the tRNA and thereby reducing the efficiency and accuracy of mitochondrial translation | [Mansergh et al. (1999)](http://www.ncbi.nlm.nih.gov/entrez/query.fcgi?cmd=Retrieve&db=PubMed&dopt=Abstract&list_uids=10090882),  [Lynn et al. (1998)](http://www.ncbi.nlm.nih.gov/entrez/query.fcgi?cmd=Retrieve&db=PubMed&dopt=Abstract&list_uids=9792552) |
|  |  |  | MERRF/MELAS overlap syndrome | G12207A  (6) | 1 |  | Ragged red fibers, significant pleomorphic mitochondrial proliferation, complex I deficiency. Mutation occurs at the first nucleotide of the 5-prime end of the molecule, which is involved in the formation of the stem region of the amino acid acceptor arm. The mutation likely affects proper processing of the precursor MTTS2 mRNA and may also affect neighboring genes. | [Wong et al. (2006)](http://www.ncbi.nlm.nih.gov/entrez/query.fcgi?cmd=Retrieve&db=PubMed&dopt=Abstract&list_uids=16950817) |
|  |  |  |  |  |  |  |  |  |
| **34** | **tRNA-Thr** | 590090 | Parkinson Disease, Susceptibility | G15950A  (4) | 1 |  | The transition affects position 70 of the aminoacyl acceptor stem of tRNA(Thr), which has been implicated as a recognition element for threonyl-tRNA synthetase and, at least in some tRNAs, in the processing of primary mitochondrial transcripts. Thus, likely to be of functional relevance and could contribute to dopaminergic nerve cell death in affected individuals | [Grasbon-Frodl et al. (1999)](http://www.ncbi.nlm.nih.gov/entrez/query.fcgi?cmd=Retrieve&db=PubMed&dopt=Abstract&list_uids=10369889) |
|  |  |  |  |  |  |  |  |  |
| **35** | **tRNA-Trp** | 590095 | Encephalopathy, mitochondrial,  Leigh Syndrome | 1-BP INS, 5537T  (8) | 2 |  | Muscle biopsy showed subsarcolemmal proliferation of mt and decreased activities of oxidative metabolism enzymes, in particular complex IV. Skeletal muscle analysis showed profound COX deficiency & complex I deficiency. | [Santorelli et al. (1997)](http://www.ncbi.nlm.nih.gov/entrez/query.fcgi?cmd=Retrieve&db=PubMed&dopt=Abstract&list_uids=9266739),  [Tulinius et al. (2003)](http://www.ncbi.nlm.nih.gov/entrez/query.fcgi?cmd=Retrieve&db=PubMed&dopt=Abstract&list_uids=12776230) |
|  |  |  | Myopathy | G5521A  (6) | 1 |  | Muscle biopsy showed a mt myopathy with many COX-negative ragged-red fibers & severe COX deficiency | [Silvestri et al. (1998)](http://www.ncbi.nlm.nih.gov/entrez/query.fcgi?cmd=Retrieve&db=PubMed&dopt=Abstract&list_uids=9673981) |
|  |  |  | Neurogastrointestinal syndrome | G5532A  (6) | 1 |  | Muscle biopsy showed COX-negative fibers and low activity of complexes I and IV. | [Maniura-Weber et al. (2004)](http://www.ncbi.nlm.nih.gov/entrez/query.fcgi?cmd=Retrieve&db=PubMed&dopt=Abstract&list_uids=15054399) |
|  |  |  |  |  |  |  |  |  |
| **36** | **tRNA-Tyr** | 590100 | Exercise intolerance and complex III deficiency | A5874G  (6) | 1 |  | Respiratory chain complex III deficiency.  Tyrosine content is highest in the cytochrome b and COX III subunits, which may explain the clinical phenotype resulting from a defect in the tRNA-tyr gene | [Pulkes et al. (2000)](http://www.ncbi.nlm.nih.gov/entrez/query.fcgi?cmd=Retrieve&db=PubMed&dopt=Abstract&list_uids=11071502) |
|  |  |  | Chronic progressive external ophthalmoplegia with myopathy, somatic | -BP DEL, 5885T  (6) | 1 |  | Functional analysis of this mutation demonstrated that even at 23% of mutant mtDNA, oxygen consumption and cell viability were decreased. This mutation alters a residue essential in the formation of the L-shaped tertiary structure of tRNA. | [Sahashi et al. (2001)](http://www.ncbi.nlm.nih.gov/entrez/query.fcgi?cmd=Retrieve&db=PubMed&dopt=Abstract&list_uids=11594340) |
|  |  |  | Chronic Progressive External Ophthalmoplegia with Myopathy, Somatic | G5877A  (6) | 1 |  | Functional analysis of this mutation demonstrated that even at 23% of mutant mtDNA, oxygen consumption and cell viability were decreased. This mutation alters a residue essential in the formation of the L-shaped tertiary structure of tRNA. | [Sahashi et al. (2001)](http://www.ncbi.nlm.nih.gov/entrez/query.fcgi?cmd=Retrieve&db=PubMed&dopt=Abstract&list_uids=11594340) |
| **37** | **tRNA-Val** | 590105 | Ataxia, progressive seizures, mental deterioration, and hearing loss | G1606A  (8) | 2 |  | G-to-A transition, affecting the acceptor stem of the mitochondrial tRNA-val. Biochemical analysis showed normal activities of respiratory chain enzymes and citrate synthase. Morphologic examination showed scattered ragged-red fibers and poor or absent cytochrome c oxidase. Magnetic resonance imaging revealed diffuse calcification of the basal ganglia and cerebral cortical atrophy | [Sacconi et al. (2002)](http://www.ncbi.nlm.nih.gov/entrez/query.fcgi?cmd=Retrieve&db=PubMed&dopt=Abstract&list_uids=12056939),  [Tiranti et al. (1998)](http://www.ncbi.nlm.nih.gov/entrez/query.fcgi?cmd=Retrieve&db=PubMed&dopt=Abstract&list_uids=9450773) |
|  |  |  | Neonatal death, Leigh syndrome | C1624T  (-) | 1 | Nuclear-encoded components or epigenetic phenomena.  This change might influence the phenotype, it probably is not a pathogenic mutation | The change was expected to affect a basepair in the dihydrouridine loop that is highly conserved in species from yeast to human. A marked, selective reduction of the steady-state level of tRNA(Val) in cardiac & skeletal muscle was found in one of the infants that died neonatally and in skeletal muscle from the mother. These and other data suggested that the mutant was rapidly degraded. The marked difference in phenotype between mother and her offspring was not explained by this defect. | [McFarland et al. (2002)](http://www.ncbi.nlm.nih.gov/entrez/query.fcgi?cmd=Retrieve&db=PubMed&dopt=Abstract&list_uids=11799391) |
