# Supplementary material for: MtSNPscore: a combined evidence approach for assessing cumulative impact of mitochondrial variations in disease
Source: BMC Bioinformatics. 2009 Aug 27;10(Suppl 8):S7. doi: 10.1186/1471-2105-10-S8-S7 (PMC2745589; doi:10.1186/1471-2105-10-S8-S7)
Supplement: Additional file 2 — Distribution of variation reported in literature across mtDNA with score summary. 2A. Gene-wise distribution of variation selected from published reports as described in text. 2B. Summary of scores assigned to these variations. Score of three, six and eight, were assigned to 24, 68 and 28 reported mutations, respectively. [file 1471-2105-10-S8-S7-S2.pdf]

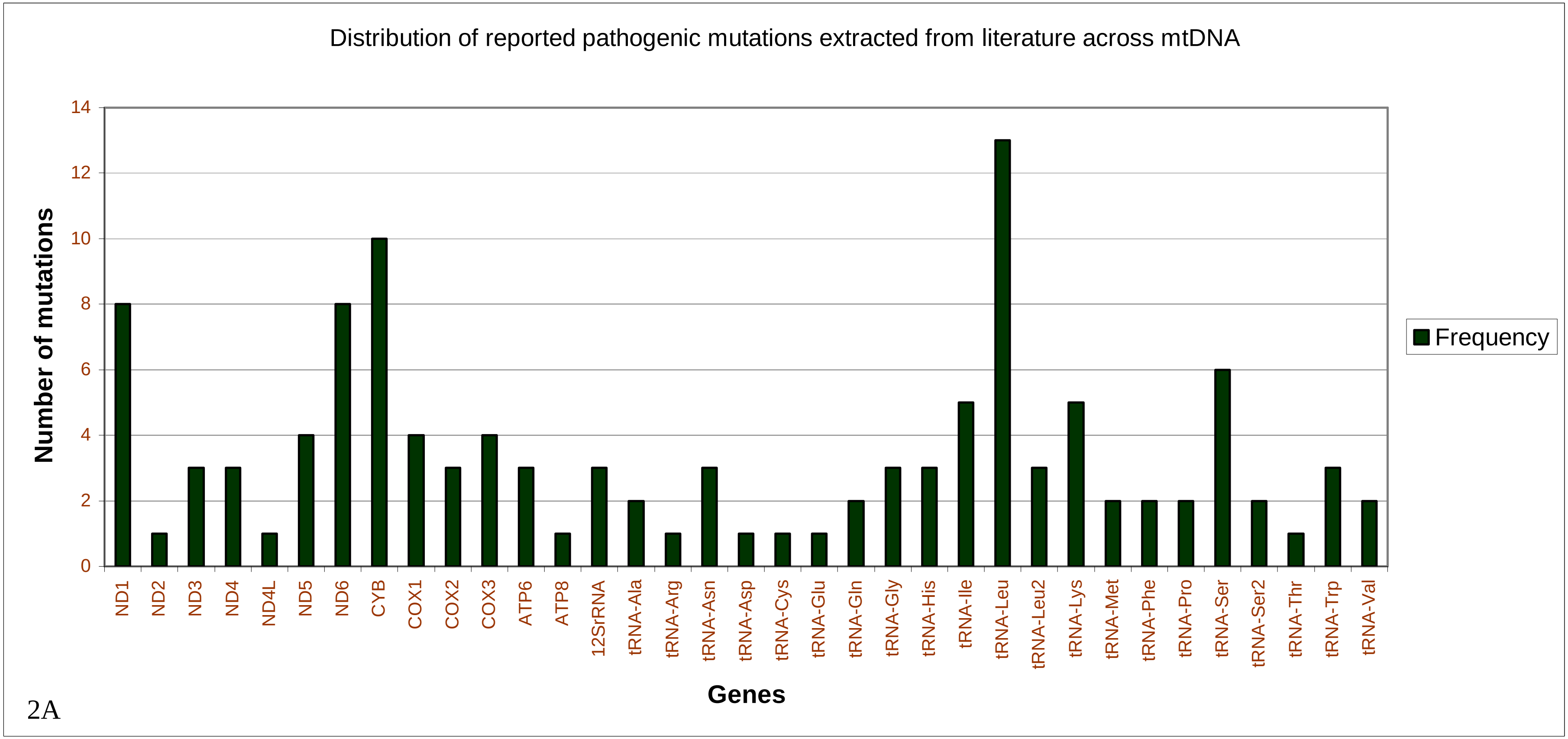

Distribution of reported pathogenic mutations extracted from literature across mtDNA
Number of mutations
0
2
4
6
8
10
12
14
ND1
ND2
ND3
ND4
ND4L
ND5
ND6
CYB
COX1
COX2
COX3
ATP6
ATP8
12SrRNA
tRNA-Ala
tRNA-Arg
tRNA-Asn
tRNA-Asp
tRNA-Cys
tRNA-Glu
tRNA-Gln
tRNA-Gly
tRNA-His
tRNA-Ile
tRNA-Leu
tRNA-Leu2
tRNA-Lys
tRNA-Met
tRNA-Phe
tRNA-Pro
tRNA-Ser
tRNA-Ser2
tRNA-Thr
tRNA-Trp
tRNA-Val
Genes
Frequency

2A

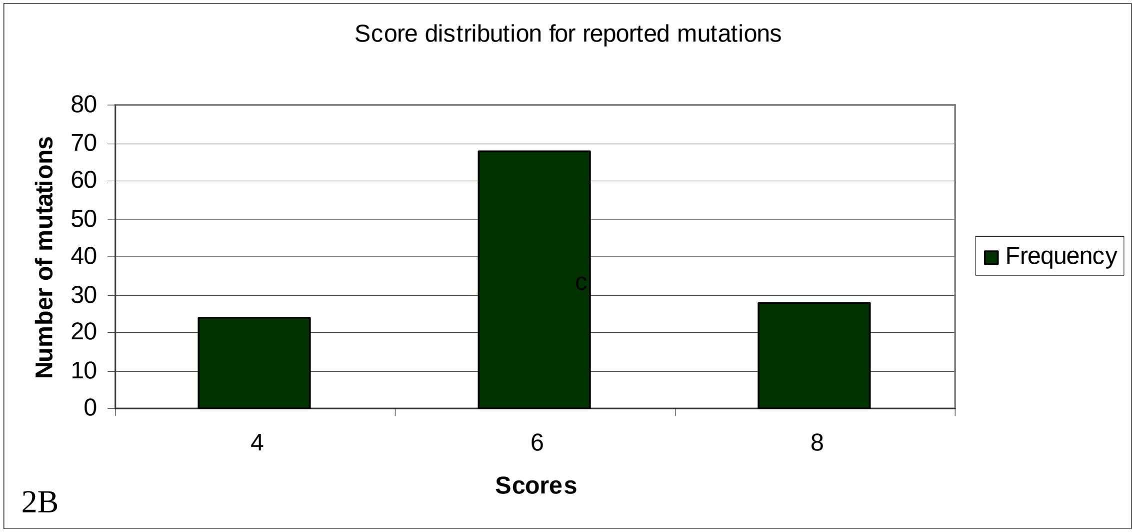

Score distribution for reported mutations
Number of mutations
0
10
20
30
40
50
60
70
80
4
6
8
Scores
Frequency

2B
