# Supplementary figures and images for: MtSNPscore: a combined evidence approach for assessing cumulative impact of mitochondrial variations in disease
Source: BMC Bioinformatics. 2009 Aug 27;10(Suppl 8):S7. doi: 10.1186/1471-2105-10-S8-S7 (PMC2745589; doi:10.1186/1471-2105-10-S8-S7)

## Summary of *in silico* prediction tools

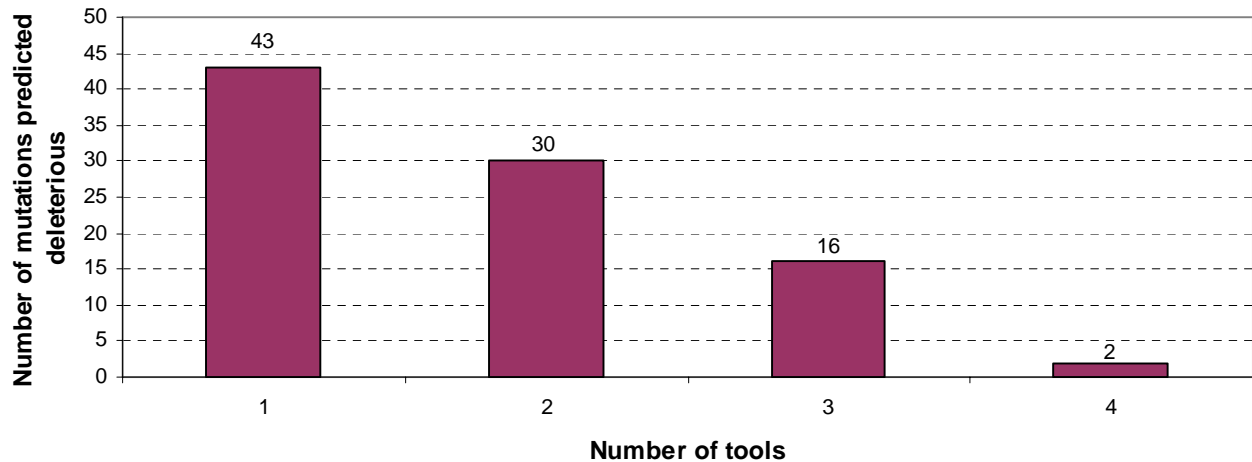

Supplement: Additional file 4 — Summary of predictions by in silico tools. Summary of predictions of reported mutations. It can be seen that all the 43 reported mutations were predicted deleterious by at least one method, followed by 30 being predicted by two, 16 by three and two by all the four in-silico methods. [file 1471-2105-10-S8-S7-S4.pdf]
